# Shrinking the Skin: Motion Results in Compressive Mislocalization of Stimuli Applied 10 s Post‐Motion

**DOI:** 10.1111/ejn.70076

**Published:** 2025-04-01

**Authors:** Tatjana Seizova‐Cajic, Jack Brooks, Janet Taylor

**Affiliations:** ^1^ Faculty of Medicine and Health The University of Sydney Sydney Australia; ^2^ Neuroscience Research Australia Sydney Australia

**Keywords:** localization bias, psychophysics, somatosensation, tactile filling‐in, tactile localization

## Abstract

Localizing touch on the skin requires integration of multiple spatial signals, including reference landmarks and motion cues. It is well known that motion patterns can bias the perceived endpoint of motion. However, it is unknown whether *static* touch presented *post‐motion* is also distorted. To investigate this, we presented space‐changing motion patterns and tested position perception 1 s and 10 s post‐motion. We used a brush moving along the forearm at 15 cm s^−1^, brushing 4.5 cm skin patches near the elbow and near the wrist, skipping a 10‐cm long metal‐shielded patch in the middle (‘numb spot’). It accelerated to 100 cm s^−1^ across the shielded gap in an attempt to create an illusion of continuous motion between the separate brushed areas. After several such deceptive motions, 12 participants indicated the locations touched by a von Frey filament near the elbow and wrist, all within the previously brushed areas. Localization responses shifted 4–10 mm towards the numb spot in the skipped‐patch condition compared with controls with either continuous brushing across the full forearm, or brushing the same patches without acceleration. This spatial distortion was equally strong 1 s and 10 s after motion offset with only isolated location‐specific differences between delays. In addition, participants' sketches indicated a reduction in perceived gap size. We propose that participants used the brushed fields as reference frame for localisation, with the high‐velocity motion compressing the perceived space between them. This means that motion‐defined boundaries can serve as spatial landmarks for static touch.

## Graphical abstract

Moving objects touch successive skin locations, providing spatial information. When a brush sweeps along the skin but rapidly accelerates to skip a patch in the middle, it creates deceptive temporal continuity between skin regions before and after the gap. This acceleration‐induced continuity causes spatial distortion: the untouched region perceptually shrinks and static touches near it are mislocalized towards it. The effect persists for at least 10 s, suggesting that stable updating of spatial relationships through motion patterns is possible.

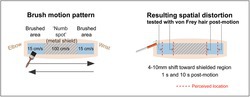

**Abbreviations**
CI: confidence interval
LMM: linear mixed model
OSF: Open Science Framework
SPSS: Statistical Package for the Social Sciences
VE: variable error

## 1 Introduction

When our skin brushes against objects, or an object sweeps across the skin, we feel both motion and *where* it is. We perceive the shape of the motion trajectory, its continuity and its location on the skin. Importantly, motion provides information about spatial order: locations touched one after another are spatially near each other. In the 19th century, Lotze proposed that the nervous system exploits this relationship—when ‘a stimulus changes its region of stimulation, the local signs change, and successive local signs are the things of adjacent localities’ (cited in Herrnstein and Boring 1965, 268). His concept of local signs bridges neural activity and spatial perception, suggesting how sequential stimulation could help establish the perceptual map of the skin.

### 1.1 Link Between Motion and Localization

This temporal–spatial link makes motion a potential spatial cue. Correlation in neural firing is an established principle in learning and map formation (Merzenich and Jenkins 1993; Wiemer et al. 2000) providing a mechanism through which motion could influence the neural basis for position perception. Supporting this idea, robust illusions show that motion biases the perceived locations of stimuli on the skin (Geldard and Sherrick 1972; Whitsel et al. 1986; Fardo et al. 2018; Seizova‐Cajić and Taylor 2014; Nguyen et al. 2016; Macauda et al. 2018; see Box 1 for details). These theoretical and empirical arguments underpin our reasoning in the present study.

Box 1 **Motion‐induced biases in position perception.**

*Basic effects of motion parameters*: High‐speed motion compresses perceived spatial intervals: in the cutaneous rabbit (sensory saltation) illusion, rapid taps (separated by approximately 100 ms intervals) at different locations appear closer together (Geldard and Sherrick 1972; Trojan et al. 2010). Similarly, continuous brushing at 100 s^−1^ makes a 4 cm distance feel half as long compared with brushing at 15 s^−1^ (Whitsel et al. 1986). Path shape affects localization of curved trajectories (Fardo et al. 2018), and the sequence of stimulation matters: reversed order of touches in the middle of a motion path degrades perception of motion direction between them (Seizova‐Cajić et al. 2020).

*Forward displacement versus compression*: Vision research introduced the concept of *motion extrapolation—*the idea that the visual system predicts future positions of moving objects to compensate for neural processing delays. This manifests as forward displacement in the direction of real or implied motion (‘representational momentum’, Freyd and Finke 1984). Neural mechanisms that may perform such extrapolation have since been discovered at multiple levels of the visual system (Hogendoorn 2020). Implied motion in touch also results in forward shift (Merz et al. 2019). However, high‐speed motion paradigms described above produce mislocalization in the opposite direction (also demonstrated by Macauda et al. 2018), showing that different motion parameters can lead to opposite spatial distortions.

*Role of predictability/anticipation*: Both within and across trials, predictability of motion also influences spatial distortions (Kerzel 2002; Merz et al. 2023). Perceived skin location of a motion pattern (cutaneous rabbit) varies dramatically with instructions about where to attend (Kilgard and Merzenich 1995). When a slowly moving stimulus suddenly accelerates across a gap and lands on the opposite side, the gap appears more compressed than when the initial motion is faster (Nguyen et al. 2016). Position shifts in motion endpoints also depend on direction and speed statistics across trials (Merz et al. 2023). These findings suggest a role for perceptual priors, and Bayesian models have been proposed (Goldreich and Tong 2013; Merz et al. 2022).

*Neural implications*: Motion‐induced position shifts may reflect processes involved in development and maintenance of cortical maps. Cortical topography changes with input statistics (Wiemer et al. 2000) through rapid disinhibition of latent inputs and longer‐term experience‐dependent plasticity (Merzenich and Jenkins 1993). If motion consistently suggests an altered skin space, corresponding map changes may follow. However, the extent to which biological neural systems use motion patterns to maintain spatial organization remains unclear.

Relative position alone is insufficient for localization, and thus, motion is insufficient to localize touch on the skin. Localization relies on integration of multiple spatial signals. It is more precise near anatomical landmarks such as wrist or elbow joints or temporary landmarks such as distinct stimuli at fixed skin locations (Cody et al. 2008; Cholewiak and Collins 2003), suggesting these are used as reference frames to determine position. Recent theoretical work suggests a process akin to trilateration, combining positions relative to multiple landmarks (Miller et al. 2022).

Although motion only provides relative position information, it clearly influences localization: even with stable anatomical landmarks present, the perceived locations of motion's start and end points show systematic biases, as studies in Box 1 demonstrate. This suggests that the nervous system combines information from both motion patterns and anatomical landmarks when determining location on the skin. However, their relative roles and temporal dynamics remain unclear.

### 1.2 Acceleration Mid‐Trajectory, Across a Gap, Distorts Perceived Positions

In our previous research on motion‐induced position shift, we capitalized on two factors known to create bias to maximize it: we used ***high speed*** known to induce spatial compression (Box 1), and built up ***speed expectation*** within and across trials (Box 1). In addition, we introduced ***a spatial gap*** in the motion trajectory to suggest that a skin patch over which the motion accelerated is not just compressed, but not even there.

**FIGURE 1:**
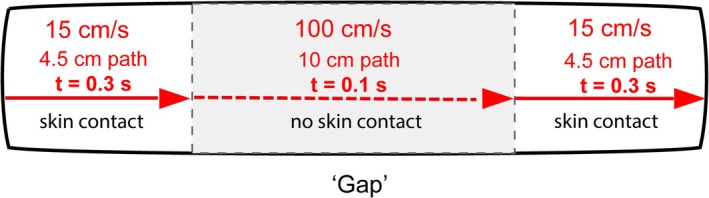
Tactile motion pattern used in our previous studies. The brush moved at 15 cm/s when touching the skin, but crossed to the other part of the skin, across the metal‐shielded patch (grey shading) in approximately 0.1 s, equivalent to the speed of 100 cm/s. For clarity, arrows indicate only one direction of motion, but the brush moved back‐and‐forth.

In those studies (Seizova‐Cajić and Taylor 2014; Nguyen et al. 2016), a brush moved along the forearm but skipped a 10‐cm patch in the middle of its path (a ‘skipped patch’). The brush moved at constant speed when touching the skin, making its motion predictable. The time required for the brush to cross the 10‐cm gap at the predicted speed was 667 ms. However, it crossed it in approximately 100 ms, equivalent to the speed of 100 cm s^−1^ (Figure 1). This motion acceleration across the gap defied the expectation and created deceptive temporal continuity between the skin locations on its opposite sides, as if the skipped patch was extremely short or did not exist.

The brush moved back‐and‐forth, in both directions. Participants indicated the perceived position of the brush *when it stopped* at different locations on the forearm on either side of the gap. Perceived positions of the stopped brush were shifted towards the gap, both when the brush halted after the skipped patch and when it halted prior to the patch. That is, the fast motion across the patch, rather than direction of motion alone or direction relative to the patch, was the key determinant of bias. These findings suggest that because locations on either side of the gap were temporally adjacent, they were perceived as spatially adjacent, based on a prior expectation of a constant velocity of brush motion.

Moreover, the magnitude of the compressive bias was greater when the brush moved more slowly on the skin, suggesting that brush motion was extrapolated based on its expected speed (skin speeds were 7.5, 15 or 30 cm s^−1^, while the speed across the gap was always the same at approximately 100 cm s^−1^; Nguyen et al. 2016).

These findings extend the literature on biases in perception of motion endpoint. However, for such biases to reflect processes involved in map maintenance, they should persist beyond the motion itself, and they should show when probed with a new and different stimulus.

### 1.3 Aim of the Present Study: To Test the Lasting Effect of Motion on Tactile Localization

We examined this by testing whether motion affects the perceived location of *subsequent stationary touches*. Previous studies typically asked participants to localize the start or end points of motion, making the motion itself the localization target, and relying on memory. Here, we applied a single‐point stimulus 1 s or 10 s after brushing the skin to determine whether the effect of motion outlasts the motion stimulus. Furthermore, the stimulus was on the skin until our participants responded to it and they did not have to rely on memory. Thus, our stimulus was different in three important ways: it was not similar to the preceding motion, it was applied post‐motion, and it did not require memory use. If anatomical landmarks alone determined its perceived location, this stimulus should be unaffected by prior motion. However, if motion serves as a spatial cue with lasting effects, systematic localization bias is expected. We used the same motion pattern as in our previous work, described in Figure 1.

Based on our previous results, we expected a *shift towards the gap* in the middle of the motion trajectory, and only *if the moving stimulus accelerated across the gap*.

## 2 Methods

### 2.1 Overview

**FIGURE 2:**
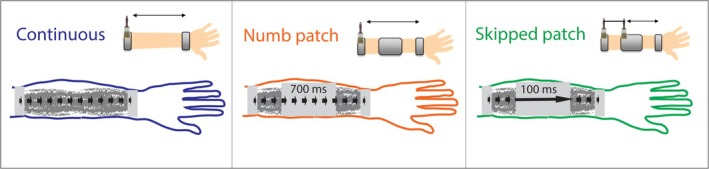
Setup and brushing patterns in three motion conditions. Continuous: the brush contacted the skin along its whole trajectory. Numb patch: the brush was in contact with 4.5‐cm skin areas flanking the numb patch while a 10‐cm metal shield prevented it from touching the middle of the forearm; it took approximately 700 ms for the brush to travel across the shield. Skipped patch: similar to the Numb patch condition, except that two rigidly connected brushes created a temporally continuous motion sweep in spite of the spatial gap present: approximately 100 ms after one brush climbed onto the shield, the other descended onto the skin on the opposite side. Note that the only difference between the Numb patch and Skipped patch conditions was the temporal gap in motion (700 ms versus 100 ms); the spatial gaps were identical.

The main experiment tested localization following brush motion along the forearm. By combining metal shields placed on the forearm with one or two brushes, we created three different motion patterns on the skin illustrated in Figure 2: (1) continuous (uninterrupted) motion; (2) motion interrupted by a 10‐cm ‘numb patch’ in the middle; (3) motion interrupted by the same numb patch, but skipped very fast, as if it did not exist. Brush speed on the skin was always the same. The brush would pause from time to time, and during the pause, we applied a single 60‐g von Frey filament to the forearm. Blindfolded participants pointed with their other arm in the direction where they felt the stimulus, by touching a graphics tablet placed alongside the target arm. They did not receive any feedback. Brushing motion would then continue. This was repeated many times for four different test locations. When all trials were completed for that session, participants sketched on a piece of paper where they felt the motion during the session.

We ran two control experiments, details of which can be found in the Supporting Information: Section 1. The first ensures that the middle part of our custom sleeve, used in two out of three conditions (see Figure 2) did not influence localization on its own, a possibility suggested by earlier findings of perceptual repulsion caused by nearby stimuli (Day and Singer 1964; Li et al. 2017). The second control experiment identified the intensity range where localization of the point stimulus remained consistent.

### 2.2 Participants

The study was approved by the University of New South Wales ethics committee. Participants gave their written informed consent. For the main experiment, 12 participants (10 female, 2 male, aged 23–43 years), all right‐handed, each attended on 3 days. Some participants were paid for their participation.

### 2.3 Apparatus and Setup

Participants were seated with their left forearm pronated and supported on an armrest placed perpendicular to the seat back. The forearm was fitted with a leather sleeve featuring two rectangular 4.5‐cm openings, separated by a 10‐cm metal shield centred on the forearm. When the brush moved across the shield, its motion could not be felt on the skin. A graphics tablet used to record localization responses was positioned vertically, medial and parallel to the forearm so that the experimenter saw the forearm, but the participant could not. Participants were blindfolded during testing.

Paintbrushes from a hardware store (0.8 cm thick × 4 cm wide, fill density ~185 filaments per/cm^2^) were used for brushing. They produced a sensation of soft touch most of the time, with some participants at times experiencing them as prickly. To achieve sufficient pressure the brushes were positioned such that they slightly splayed, resulting in an effective thickness greater than 0.8 cm. They were mounted on a carrier driven along a slider at 15 cm s^−1^ by a stepper motor (Excitron Au Controller coder model Au57‐40 M) controlled using a custom program written in Labview. A smooth 15 cm s^−1^ motion was achieved by steeply accelerating the stepper motor at the start and end of the movement.

### 2.4 Experimental Conditions

The main independent variable in the experiment was motion pattern as described earlier and illustrated in Figure 2, with three conditions labelled **Continuous**, **Numb patch** and **Skipped patch**. They were presented on separate days, in counterbalanced order. Other manipulated variables, further described below, were target location on the skin, delay in localization and direction of the last sweep.

The primary dependent variable was localization response. Perceived position was tested at locations on either side of the numb patch using a single 60‐g von Frey filament. Importantly, targets were presented *after* motion offset. The secondary dependent variable was perceived location of brushed area(s), reported by shading the relevant area(s) on an outline of the forearm we provided (examples are shown in Figure 8).

### 2.5 Motion Stimuli

Brush speed on the skin was 15 cm s^−1^ in all conditions. When it moved across the metal shield, brush motion could not be felt on the skin.

In Continuous and Numb patch conditions, a single paintbrush moved up and down the arm, while two brushes were used in the Skipped patch condition, as shown in Figure 2. In the Continuous condition, participants wore a leather sleeve with a 19‐cm window over the dorsum of the forearm, flanked by one narrow metal shield near the elbow and one near the wrist (Figure 2, left panel). In Numb patch and Skipped patch conditions, only two 4.5‐cm skin patches were exposed to brushing (Figure 2, central and right panels). Each sequence of sweeps along the forearm began and ended on the metal shield near the elbow and wrist.

In the Skipped patch condition, two brushes were used but only one was in contact with the skin at any given time. Distance between the outer edges of the two brushes was approximately 10 cm, equal to the shield extent. This resulted in approximately 0‐ms temporal gap between offset of stimulation on one side of the gap and its onset on the opposite side, or slightly longer (approximately 100 ms) for the full strength of the stimulus to transfer from one side to the other. This time gap varied slightly trial‐to‐trial and between participants due to variation in brush splay. Using 100 ms as the estimate of this temporal gap, the brush speed was 100 cm s^−1^ across the shield, compared with 15 cm s^−1^ across the skin it contacted.

In Continuous and Numb patch conditions, one sweep from elbow to wrist (or vice versa) lasted just over 1.3 s (including the time to move on and off the metal shields). In the Skipped patch condition, it lasted only 0.7 s, because the brush took less time to cross the gap.

During localization testing, the brush was driven off the skin and rested on the metal shield at one of its ends (elbow rest or wrist rest) and in the middle (when two brushes were used).

### 2.6 Touch (Localization) Stimuli

**FIGURE 3:**
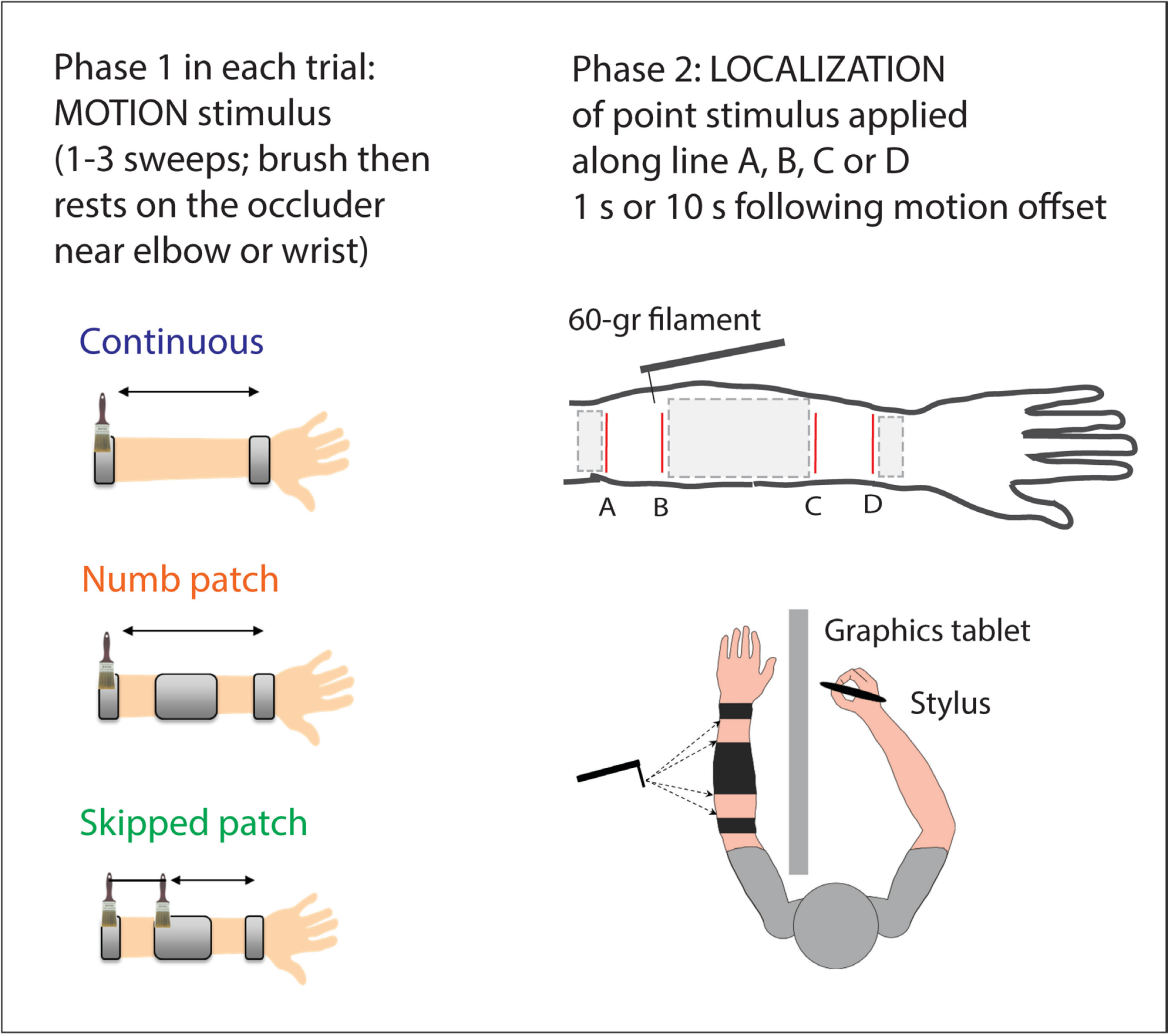
Trial structure in motion conditions. Motion stimulation preceded the localization task. The arm was occluded from view by a graphics tablet and a cloth (not shown), and participants responded using a stylus.

The localization target was a 60‐g von Frey filament (also known as Semmes–Weinstein monofilament). The filament bends when this force is reached, providing inherent control over the applied pressure. The value was selected after the second control experiment (see Supporting Information). It was manually applied by the experimenter at locations next to the numb patch (0.5 cm from its border) or further away (4.5 cm from its border). Labelled A, B, C and D, the respective locations were 9.5 and 5.5 cm proximal, and 5.5 cm and 9.5 cm distal to the centre of the forearm. Four thin lines were drawn on the forearm and the experimenter applied the point stimulus anywhere along the line (Figure 3, right panel). As the experimenter sat to the left of the participant, any inadvertent skin stretch signal was likely in a medio‐lateral direction.

The filament was applied approximately 1 s or 10 s after motion offset. The longer delay was used to examine if shift in perceived position persists. A counter displayed on the experimenter's screen started when brush motion stopped, helping regulate the timing of von Frey stimulation at the designated delays. While timing variation was minimized through this system, some small variation was inherent due to manual application. Participants were asked to respond immediately upon feeling the stimulus.

### 2.7 Procedure

#### 2.7.1 Localization Task

When the experimenter applied the von Frey filament, participants pointed with a stylus held in the right hand to indicate on the graphics tablet where they perceived the touch on the skin on the other side of the tablet (in orthogonal direction). The stimulus was removed after pointing. This position was recorded via the graphics tablet placed in between the two arms, meaning that participants did not touch the skin. They also did not receive other form of feedback.

In motion conditions, localization task was performed 1 s or 10 s following motion sweeps (illustrated in Figure 3; details are explained below). Participants were asked to respond immediately.

#### 2.7.2 Experimental Sequence

Each participant attended three sessions, and each session consisted of three blocks of trials: Baseline position tests, Position tests interleaved with motion, and Post‐test. After the three blocks, participants completed a phenomenological report about the motion they experienced in the middle block. Example sequence for a single participant is shown in Table 1.

Block 1: **Baseline position tests** At the start of each session, the von Frey stimulus was presented in random order at four locations, eight times each, for a total of 32 trials (4 locations × 8 repeats). Localization stimuli applied in this condition were not preceded with motion.
Block 2: **Position tests interleaved with motion** In the brushing conditions (Continuous, Numb patch and Skipped patch) each trial consisted of 1–3 brushing sweeps (‘conditioning’), followed by the application of 60‐g von Frey filament, and a pointing response. The filament was applied approximately 1 s or 10 s after motion offset; this was subject to small variation because of manual application. Participants received two practice trials. Subsequent testing consisted of 128 trials (4 locations [A, B, C, D] × 2 delays [1 s, 10 s] × 2 directions of the last sweep [proximal, distal] × 8 repeats), delivered in two blocks separated by a short break.

**TABLE 1:** Example experimental sequence.

Session 1 (Day 1)	Baseline (32 trials)	Continuous motion interleaved with position test (128 trials, preceded by two practice trials)	Post‐test (32 trials)	Phenomenological report (a drawing)
Session 2 (Day 2)		Numb‐patch motion interleaved with position test (128 trials, preceded by two practice trials)		
Session 3 (Day 3)		Skipped‐patch motion interleaved with position test (128 trials, preceded by two practice trials)		

*Note:* The middle column presents one possible order of experimental conditions; the order was randomized across participants.

*Conditioning*: We chose short conditioning to maximize the number of trials, expecting that any cumulative effects would occur across trials. The average number of forearm sweeps delivered at each target location was approximately 2 (elbow‐wrist, EW, followed by wrist‐elbow, WE, or vice versa), with 1, 2 or 3 sweeps applied in each trial. The brush starting position (elbow or wrist) and direction of approach to target location (proximal or distal) were counterbalanced at each target location. This influenced the number of required sweeps. For example, when the brush started from the elbow, and direction was supposed to be distal, that required 1 sweep (EW) or 3 sweeps (EW, WE, EW), while proximal direction required 2 (EW, WE). Starting position, target location and direction of approach were randomized across trials, and frequency of 1, 2 and 3 sweeps was approximately equal.

Block 3: **Post‐test** A 32‐trial post‐test was identical to the Baseline (Block 1).

**Phenomenological report**: At the end of the session, participants indicated *where* they had felt the brushing during the session by drawing on a template. The template page contained three forearm outlines to allow for three different drawings in case their perception changed during the brushing session.

### 2.8 Data Analysis

The outcome variable was position of the response on the tablet measured along a single dimension, from the origin that was assigned a value of zero. Larger values represent more distal locations (further away from the body). In this section, we explain how these raw data were used to compute the variable error and constant error. We also analysed phenomenological reports, described last.

#### 2.8.1 Position Uncertainty in Localization Responses (Variable Error)

Variable error (VE) of pointing responses was computed separately for each participant as unsigned (absolute) deviation from the mean of their repeated responses to the same target (A, B, C or D) in the same experimental condition. There were eight repeats per target location in each condition. Baseline had 24 repeats per location altogether, completed across three sessions on separate days, and its VE was based on 8 repeats within the same session. We compared VE in different conditions and target locations using linear mixed model (LMM). Location was treated as a continuous variable (−9.5, −5.5, 5.5, 9.5), and it was a fixed factor along with motion condition (Continuous, Numb patch, Skipped patch) and Delay (1 s, 10 s). We evaluated quadratic trend across tested locations. Participants were the random factor. The outcome variable was the absolute value of individually centred responses. One per cent of most extreme data points in each condition was removed. All results were averaged across the control variable direction of the last sweep.

#### 2.8.2 Position Shift in Localization Responses (Bias, Constant Error)

To prepare the data, we (a) pooled the responses from three Baseline sessions and (b) centred the data within each condition.

Each participant completed one Baseline run on each of the three testing days, and we averaged their values across the 3 days. Specifically, we averaged trials number 1 from each day for a pooled trial 1 response, and likewise for repeats 2–8. This resulted in pooled Baseline data with 8 ordered repeats per participant per location. We used the pooled data in the subsequent analyses.
To eliminate distribution‐level shifts that may occur across sessions and contaminate direct comparisons of responses across sessions (Brooks et al. 2019), we centred the data. We subtracted each response from the participant's mean in each experimental condition/session. Accurate responses would produce values of −9.5, −5.5, 5.5 and 9.5 cm for targets A, B, C and D, respectively. Naturally, our observers made errors, which we analysed in the next step to determine if there was systematic bias.

We analysed motion‐induced position shift using linear mixed model (LMM). All pairwise comparisons were conducted with Bonferroni correction. Details of the analyses are given below and summarized in Table 2. The SPSS files with models and outputs are available on OSF (Open Science Framework).

**TABLE 2:** Summary of LMM analyses of constant error (position shift).

	Predictors	Target location treated as …	Outcome variable	Accurate values for target locations A, B, C and D
LMM Preliminary analysis (see Supporting Information: Section 3)	Condition (Base, Cont., NP, SP) Target location (A, B, C, D) Trial order (1–8) (+ interactions)	Continuous variable	Signed value of individually centred responses	Signed −9.5; −4.5; 4.5; 9.5
LMM Analysis 1	Condition (Base, Cont., NP, SP) Target location (A, B, C, D) (+ interaction)	Categorical variable	Absolute value of individually centred responses	Absolute 9.5; 4.5; 4.5; 9.5
LMM Analysis 2	*For each Condition separately:* Target location (A, B, C, D) Delay (1 s, 10 s) (+ interaction)			
LMM Analysis 3	Condition (Base, Post‐test Cont, Post‐test NP, Post‐test SP) Target location (A, B, C, D) Trial order (1–8) (+ interactions)			

Abbreviations: *Base*, Baseline; *Cont*., Continuous; *NP*, Numb Patch; *SP*, Skipped Patch.

LMM Preliminary analysis (reported in the Supporting Information ): Effect of Motion condition, Location and Order of repeats on perceived position Here, we treated Location as continuous variable to simplify the model, which allowed us to include Order of repeats in the analysis. However, this forced estimates of responses to all locations onto a single function, which might not have been justified. Locations were thus allowed to vary independently of each other in the main analysis (LMM Analysis 1). This produced very similar results.

LMM Analysis 1: Effect of Motion condition and Location on perceived position This was our main analysis, with Motion condition (Baseline, Continuous, Numb patch, Skipped patch) and Location of the test (A, B, C, D) as fixed factors. Two‐way interaction was also included. Target location was treated as a categorical variable. To allow us to test the main effect of condition across all locations, the outcome variable here was the *absolute* value of the centred response. Perfectly accurate responses would produce 9.5, 5.5, 5.5 and 9.5 cm for targets A, B, C and D, respectively, and compressive shift at any location would result in smaller value compared with Baseline (Baseline shows a compressive shift itself). Order of repeats was omitted from this analysis because it showed no trend in the preliminary analysis. Delay (1 s, 10 s) was also omitted because we wanted to include Baseline, which had no delay; the effect of delay was analysed separately in Analysis 2.

LMM Analysis 2: Effect of delay between motion offset and application of target stimulus Similar in format to Analysis 1, this analysis tested the effect of delay for each motion condition separately to avoid complex interactions of no interest to us. Fixed factors were Location (A, B, C, D) and Delay (1 s, 10 s). The outcome variable was the absolute value of centred responses.

LMM Analysis 3: Effect of Motion condition on Post‐tests: Post‐tests conducted following each motion condition (Continuous, Numb patch, or Skipped patch) were compared with Baseline to determine whether any position shifts persisted. This analysis was similar to the LMM Analyses 1 and 2, treating Location as categorical variable. The outcome variable was the absolute value of centred responses.

The responses were averaged across Direction of motion, our controlled variable.^1^

#### 2.8.3 Phenomenological Report

Participants used between one and three forearm templates to sketch their perception of motion. Individual drawings can be viewed on OSF. To compare it across conditions, we expressed perceived gap size in each drawing as proportion of end‐to‐end motion extent in the same drawing. Where participant created more than one drawing, the average value was used.

To analyse perceived intensity from participants' drawings, each scanned forearm template was divided into 10 equal segments along its length. A typical forearm was 25–30 cm long and each segment represented 2.5 to 3 cm of forearm length. The pixel darkness value was measured for each segment (0 = white, 255 = black), providing a quantitative measure of shading intensity. This allowed us to map perceived intensity along the forearm while preserving individual variation in how participants chose to represent intensity through shading. While participants likely varied in their mapping of sensation intensity to shading darkness, this analysis enabled within‐subject comparisons across conditions and systematic assessment of intensity patterns along the forearm.

## 3 Results

Data files and all the analyses are available on OSF.

### 3.1 Key Findings

Linear mixed model analysis revealed that acceleration across the gap systematically distorted the perceived spatial layout of the forearm. The key findings were as follows:

1. An apparent compression of perceived space in the Skipped patch condition, where high‐velocity motion connected regions across the gap. This compression was approximately 10% at each tested location relative to Baseline (9%, 11%, 8% and 10% for locations A, B, C and D respectively), with significant differences at three out of four locations (*p* = 0.0001, 0.039, and 0.0001 for locations A, B and D).
2. Neither Continuous nor Numb patch conditions showed significant deviations from Baseline. The compression was specific to the Skipped patch condition. LMM analysis showed a significant interaction between Condition and Location (*F*(9, 642.7) = 2.35, *p* < 0.013), with post‐hoc tests confirming that only the Skipped patch condition differed from other conditions.
3. The compression effect remained stable over the course of 10 s: there was no significant difference between the mean response at 1 s versus 10 s delays in the Skipped patch condition (mean difference = 0.02 cm, *p* = 0.87).
4. Participants' drawings confirmed this pattern: the perceived gap between brushed areas was smaller in the Skipped patch condition (31% of total motion extent) compared with the Numb patch condition (52%), while no gap was reported in the Continuous condition. This difference in perceived gap size between Skipped and Numb patch conditions was significant (mean difference = 21%, *p* = 0.049).

### 3.2 Position Uncertainty (Variable Error, VE)

**FIGURE 4:**
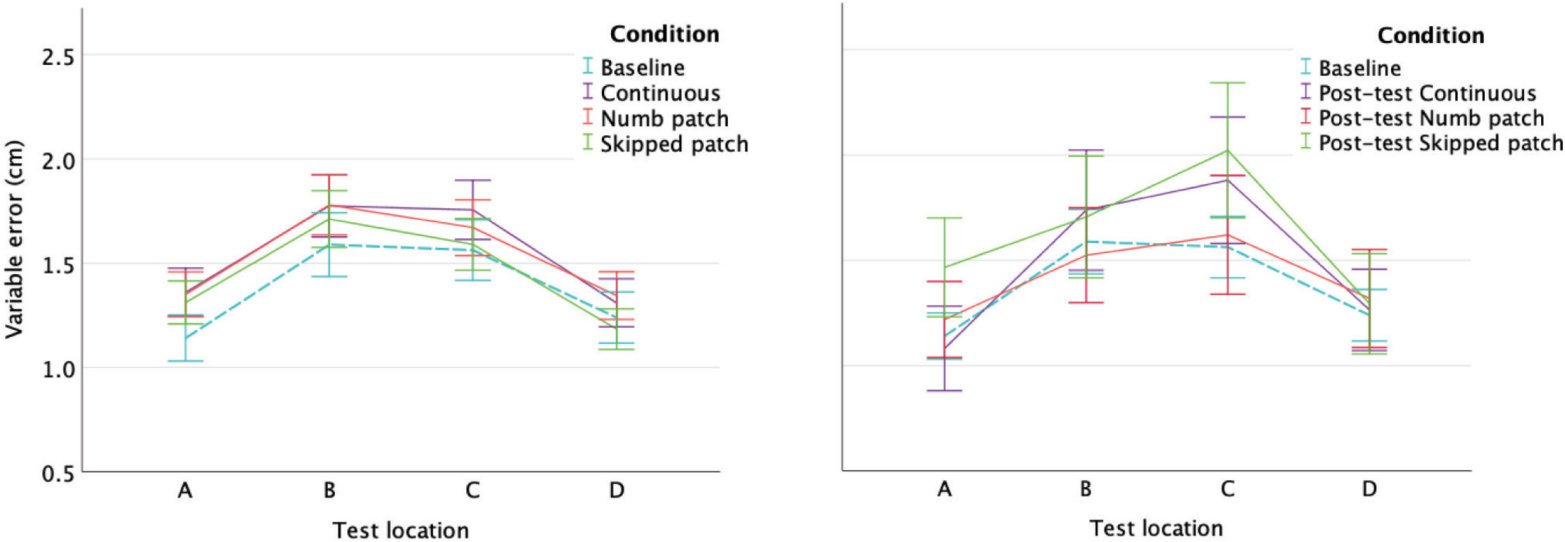
Position uncertainty of individual responses at each location in Motion conditions (left panel) and Post‐test (right panel); Baseline is shown in both panels (dashed line). Error bars are 95% CIs around means. Localization responses are more variable in the middle of the forearm than near its ends. (For individual plots, see Supporting Information: Section 2; Figure S4).

To understand how motion patterns affected localization, we first examined the uncertainty in participants' responses. Position uncertainty in localization responses is greater in the middle of the forearm than near elbow or wrist in all experimental conditions, including Baseline (see Figure 4). Motion conditions with both delays (1 s, 10 s) and post‐tests both show this pattern, as do most individual data (see, Supporting information Figures S3, S4 and S5).

LMM for variable error in motion conditions (Figure 4, left) shows a significant quadratic trend across the four locations (*F*(1, 10.97) = 22.453; *p* = 0.001) and a three‐way interaction between Motion condition, Location and Delay (*F*(3, 1130.65 = 3.717; *p* = 0.011): quadratic trends across locations are slightly different at 1 s delay compared with 10 s in different motion conditions, as shown in Figure S5. Typically, variable error for locations near anatomical landmarks (wrist and elbow) is lower 10 s post‐motion compared with 1 s. No other main effect or interaction was significant.

A separate LMM was conducted on Post‐test data (Figure 4, right). Quadratic trend for Location was again significant (*F*(1, 11) = 18.47; *p* = 0.001, and so was the main effect of Condition (*F*(1, 1259) = 3.174; *p* = 0.024). Paired comparisons with Bonferroni correction show the significant difference between Baseline (mean VE = 1.83 cm) and Post‐test Skipped patch (2.05 cm). Neither of them was significantly different from Post‐test Continuous (1.89 cm) or Post‐test Numb patch (1.87 cm). Interactions were not significant and were not included in the final model.

The residuals in both analyses show some positive skew.

### 3.3 Position Shift (Constant Error, Bias)

Having established the pattern of uncertainty, we next examined systematic biases in localization*—*specifically, the compression effect highlighted above. The following analyses compare localization responses across conditions and target locations and show that compression occurred only in the Skipped patch condition.

#### 3.3.1 LMM Preliminary Analysis

Location was in this analysis treated as a continuous variable, thus constraining the estimates for all locations within each condition to a single function. Given that data were centred around individual means in each condition, the main effect of Condition was not statistically significant (*F*(3, 380) = 0.077; *p* = 0.972). Location was significant (*F*(1, 11.0) = 419.0; *p* < 0.001), and so was the interaction between Condition and Location (*F*(3, 369.0) = 17.59; *p* < 0. 001). Other interactions were omitted from the final model because they did not improve it.

**FIGURE 5:**
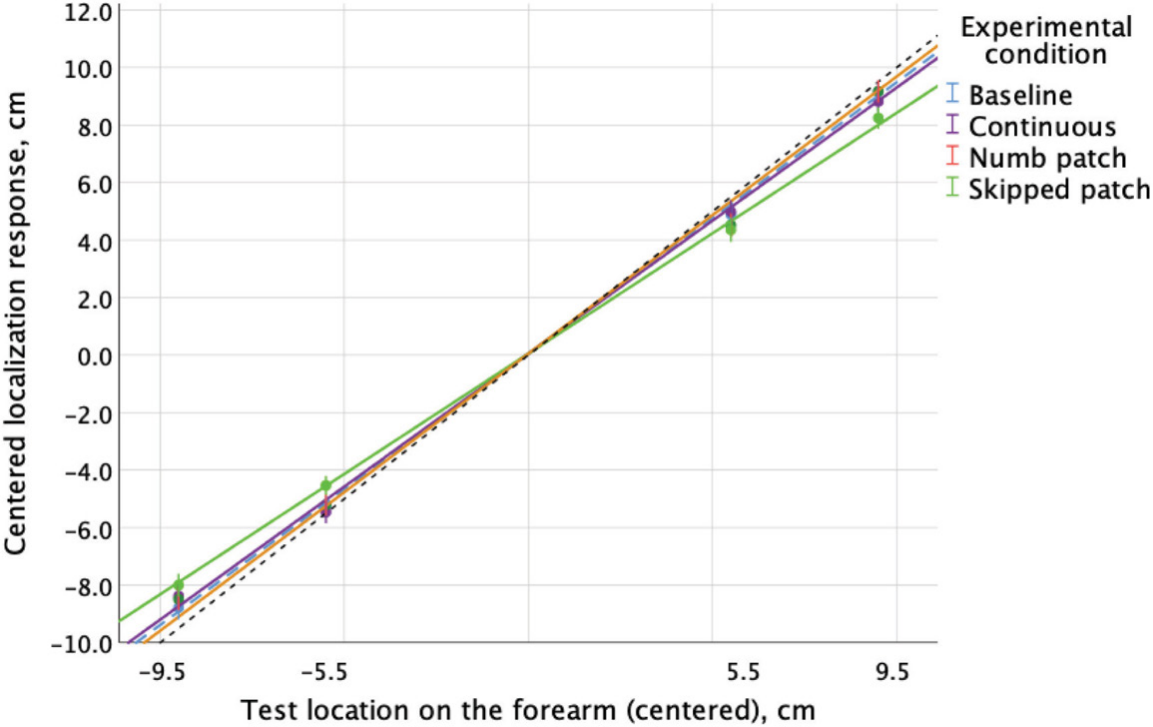
Localization response as a function of test location on the forearm and motion condition. Individual points are means based on responses of 12 participants who each contributed 32 responses per data point (in motion conditions: 2 directions of the last sweep × 2 delays × 8 repeats; in Baseline: 32 repeats). Black dotted line is a reference with the slope of one (perfectly accurate responses), while Baseline is shown as the dashed blue line. Continuous and Numb patch conditions are similar to Baseline, while Skipped patch—the green line*—*shows the greatest compression. Regression lines are based on the LMM Preliminary analysis.

The main result of interest here was the difference in slope for different conditions (Figure 5; regression coefficients are displayed in Table 3). All conditions show compression (inward shift) relative to target locations, and Skipped patch is the most compressed. Pairwise comparisons with Bonferroni correction show that it significantly differed from other motion conditions and from the Baseline at all locations (p values to three decimal places range from 0.000 to 0.033), apart from the Continuous condition at the −5.5 and + 5.5 target locations. No other comparisons between any of the conditions were statistically significant. Order of repeats did not show consistent trend. It was initially included in the analysis but omitted from the final model.

**TABLE 3:** Localization response (y) as a function of Motion condition and Location (x).

Motion condition	Predicted y =
Baseline	0.051 + 0.944 x
Continuous	0.067 + 0.910 x
Numb patch	0.087 + 0.928 x
Skipped patch	0.037 + 0.852 x

More details can be found in Supporting Information, Section 3, including mean estimates based on the model (Table S1), and a figure showing responses as a function of target location and order of repeats (Figure S6).

#### 3.3.2 LMM Analysis 1: Effect of Motion Condition and Location on Perceived Position

**FIGURE 6:**
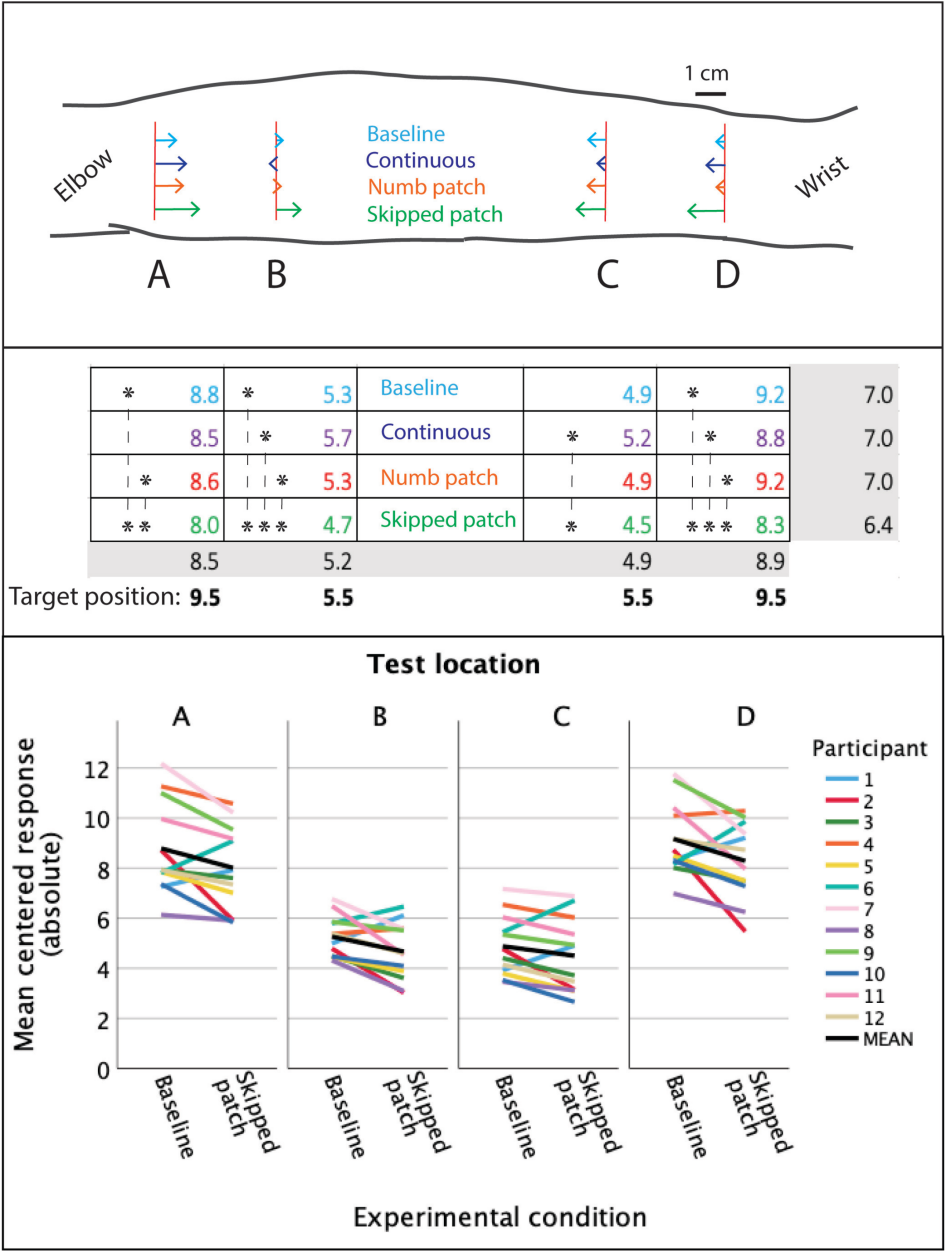
Shift in perceived position relative to target location (shift was measured along the longitudinal dimension, orthogonal to the target line). Top: Arrows indicate position shift in each of the four conditions. Values were estimated using linear mixed model (LMM Analysis 1) and estimated marginal means are shown to scale. A 1‐cm line is shown for reference. Middle: Same data, absolute values, with asterisks indicating statistically significant differences between conditions (p <.05), Bonferroni‐corrected. At every location, only Skipped patch was significantly different from other conditions. No other differences were significant. Values in grey cells are marginal means. Target positions at the bottom are distances of target locations (red lines in the drawing) from the center of the pattern. Bottom: Individual data showing that Skipped patch is compressed compared with Baseline in majority of participants (9 to 10 out of 12) at every location. (For individual plots covering all conditions, see Supporting Information, Section 4, Figures S7 and S8).

While the preliminary analysis provided meaningful insights, it constrained position shifts across locations to follow a linear pattern. In our main analysis (LMM Analysis 1), we treated Location as a categorical variable to allow for potential non‐linear effects at each target location. Figure 6 illustrates these results, with arrows showing the magnitude and direction of position shifts for each condition to scale. Individual data for Skipped patch compared with Baseline (Figure 6, bottom) shows consistent compression across most participants and all locations (A–D). Figures S7 and S8 show individual data patterns across all conditions.

The LMM analysis revealed a significant interaction between Condition and Location (*F*(9, 642.7) = 2.35, *p* < 0.013). Post‐hoc comparisons with Bonferroni correction showed that only the Skipped patch condition differed significantly from Baseline. In this condition, compression was remarkably consistent across locations, showing approximately 10% shift towards the centre compared with Baseline (9%, 11%, 8% and 10% for locations A, B, C and D respectively; *p* = 0.0001, 0.039, 0.349, and 0.0001; see Figure 6 middle and bottom panels). The other conditions showed no significant shifts relative to Baseline. The left half of Table 4 presents detailed comparisons of all conditions, expressed as deviations from Baseline values.

**TABLE 4:** Localization responses (in cm) relative to Baseline, by forearm location (A: near elbow; D: near wrist) and experimental condition.

	During motion conditioning				In post‐test			
Condition	Location				Location			
	A	B	C	D	A	B	C	D
Continuous	0.33	−0.46	−0.31	0.35	−0.32	−0.04	−0.30	−0.13
Numb patch	0.23	−0.03	0.01	−0.01	−0.22	−0.34	−0.20	−0.43
Skipped patch	0.77*	0.59*	0.38	0.88*	0.19	0.36	0.10	0.17

*Note:* Asterisks indicate statistically significant deviations from Baseline (*p* < 0.05). Skipped patch condition produced the greatest shift. Values represent estimated marginal means from LMM Analysis 1. For more details, see OSF.

#### 3.3.3 LMM Analysis 2: Effect of Delay Between Motion Offset and Application of Target Stimulus

The von Frey filament was applied either at 1 s or 10 s following motion cessation. Table 5 presents discrepancies in localization responses between these two delays. Baseline is not included because it was not preceded by motion. Each motion condition was tested separately, and all comparisons were subject to Bonferroni correction. A significant Delay by Location interaction was found in all three motion conditions (p‐values of 0.019, 0.013 and 0.002 in Continuous, Numb patch and Skipped patch conditions, respectively). These interactions occurred because at one location in each condition, 1‐s and 10‐s delays differed: in Continuous, at location D (greater inward shift at 1 s than at 10 s, *p* = 0.016); in Numb patch, at C (greater inward shift at 1 s, *p* = 0.007), and in Skipped patch, at B (greater inward shift at 10 s, *p* = 0.003). No other comparison was statistically significant. Overall, Delay had no consistent effect.

**TABLE 5:** Differences between localization responses depending on Delay (1 s, 10 s).

		Location (and absolute value of ideal response in cm)			
Condition	Difference	A	B	C	D
Continuous	10 s − 1 s	0.08	−0.35	−0.12	0.45*
Numb patch	10 s – 1 s	0.34	−0.32	0.51*	0.29
Skipped patch	10 s − 1s	0.29	−0.55*	0.05	0.28

*Note:* Positive values represent greater shifts towards the middle of the forearm after 1‐s delay. Values derived from estimated marginal means in LMM Analysis 2. Asterisks mark significant differences. For more details, see OSF.

#### 3.3.4 LMM Analysis 3: Effect of Motion Condition on Post‐Tests

**FIGURE 7:**
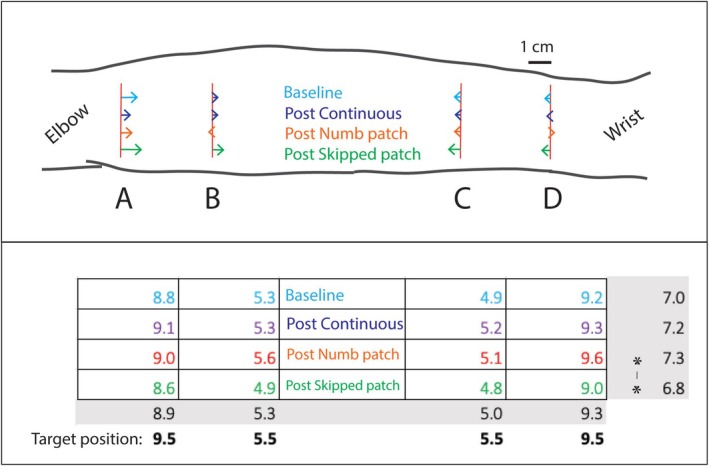
Shift in perceived position relative to target location in Post‐tests, same format as Figure 6. Estimated marginal means from LMM Analysis 3 are shown to scale. None of the comparisons within individual locations were statistically significant, but the main effect of motion condition was, due to the inward shift in the Post‐test after Skipped patch relative to Numb patch, as indicated by an asterisk (5 mm difference; *p* = 0.015).

In each session, we administered the same von Frey test stimuli at the end of the session that were initially presented at the Baseline. The responses are shown in Figure 7 along with responses to Baseline. The main effect of condition was significant (*F*(3, 489.7) = 3.61; *p* = 0.013). The Skipped patch showed 5 mm average compression across all locations compared with the Numb patch; this difference was statistically significant (*p* = 0.015, with Bonferroni correction). However, neither of the two significantly differed from the Baseline, and no other significant differences were found between the motion conditions. As expected, the main effect of location was significant (*F*(3, 668.0) = 494.3; *p* < 0.001). There was no interaction between motion conditions and location (*F*(9, 668.0) = 0.208; *p* = 0.993).

### 3.4 Phenomenological Report

Participants' drawings provided an independent confirmation of the compression effect and revealed how motion patterns influenced perceived intensity of touch.

**FIGURE 8:**
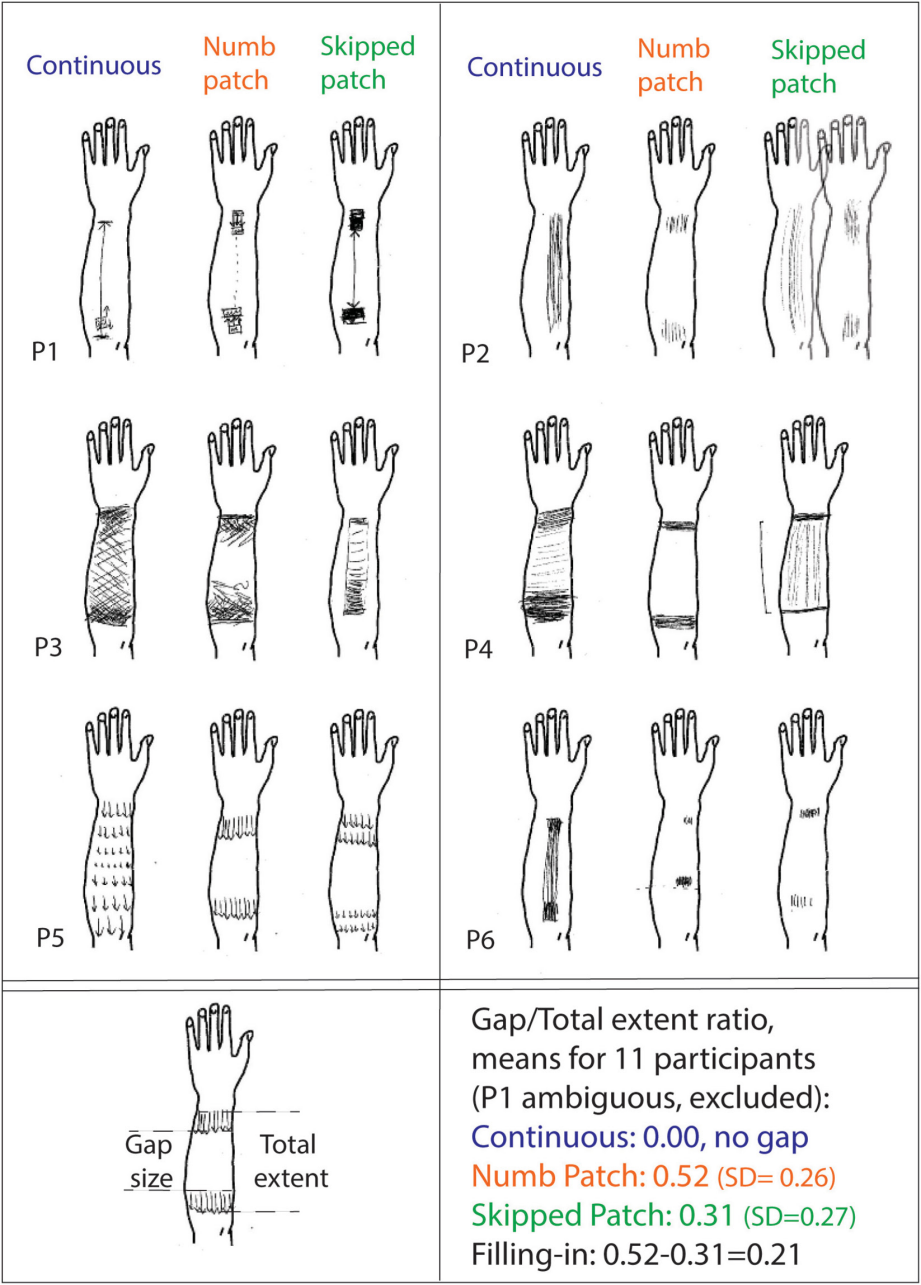
Phenomenological reports collected at the end of each session. Top panel: Drawings from the first 6 out of 12 alphabetically ordered participants (see the Supporting Information for a complete set of drawings). Participants were asked to draw ‘what they felt’, with darker regions indicating more intense brushing. Continuous motion trajectory was experienced as such; the brushing in the Numb patch often felt interrupted, while the Skipped patch varied—some participants felt it as continuous, while others did not. Participants were allowed to complete more than one drawing to report more than 1%. Bottom left: for 11 participants, we calculated the gap to motion ratio from the drawings, averaging when multiple drawings were provided per condition (see P2, Skipped patch, for an example). Bottom right: Mean ratios. Results show the Skipped patch typically lead to a perceptual ‘filling‐in’ compared with the Numb patch.

Drawings were made at the end of each session*—*Continuous, Numb patch or Skipped patch—to illustrate where participants felt the brushing, and how intensely. Most participants created one drawing to represent their sensory experience, although a few produced two or three drawings per session to depict the evolution of their perception throughout the session. One drawing for each condition from six participants is shown in Figure 8 (for the full record of all drawings, see OSF).

The gap size and total motion extent were measured in each image, as illustrated in the bottom left panel of Figure 8. Gap size was then expressed as a proportion of total motion for each drawing. The corresponding mean proportions are shown in the bottom right panel for the three motion conditions. Our measure of filling‐in was the difference between the mean gap size in Numb patch and Skipped patch conditions (0.52–0.31 = 0.21).

In the Continuous condition, the common experience among all participants was uninterrupted motion along the forearm, as reflected by a mean gap size of zero (no perceived gap). In 7/12 drawings, a denser shading around the elbow and wrist regions indicates stronger perceived intensity of brushing in those areas. Some participants*—*see P1 in Figure 8 *—*chose to use a single line to convey movement along the forearm. It remains ambiguous if this line was also meant to communicate that perceived intensity of the brushing was low.

In the Numb patch condition, 75% of participants*—*9 out of 12*—*reported experiencing a gap throughout the session. Of the three participants who did not, one initially felt the gap, but as the session progressed, it seemed to fill in and they no longer perceived it. Another participant reported continuous motion throughout. The third report was ambiguous: P1 in Figure 8 used a dotted line in the Numb patch condition to link two areas where touch was strongly felt. The average gap size in the Numb patch condition, computed across 11 participants (excluding the ambiguous report of P1) was 0.52 (95% CI: 0.34–0.70).

In the Skipped patch condition, 4 participants felt the gap throughout the session, 3 never felt it, while 4 experienced it only intermittently, at times feeling continuous or nearly continuous motion. The average gap size computed as a proportion of total motion across 11 participants, excluding P1, was 0.31 (95% CI: 0.13–0.49).

**FIGURE 9:**
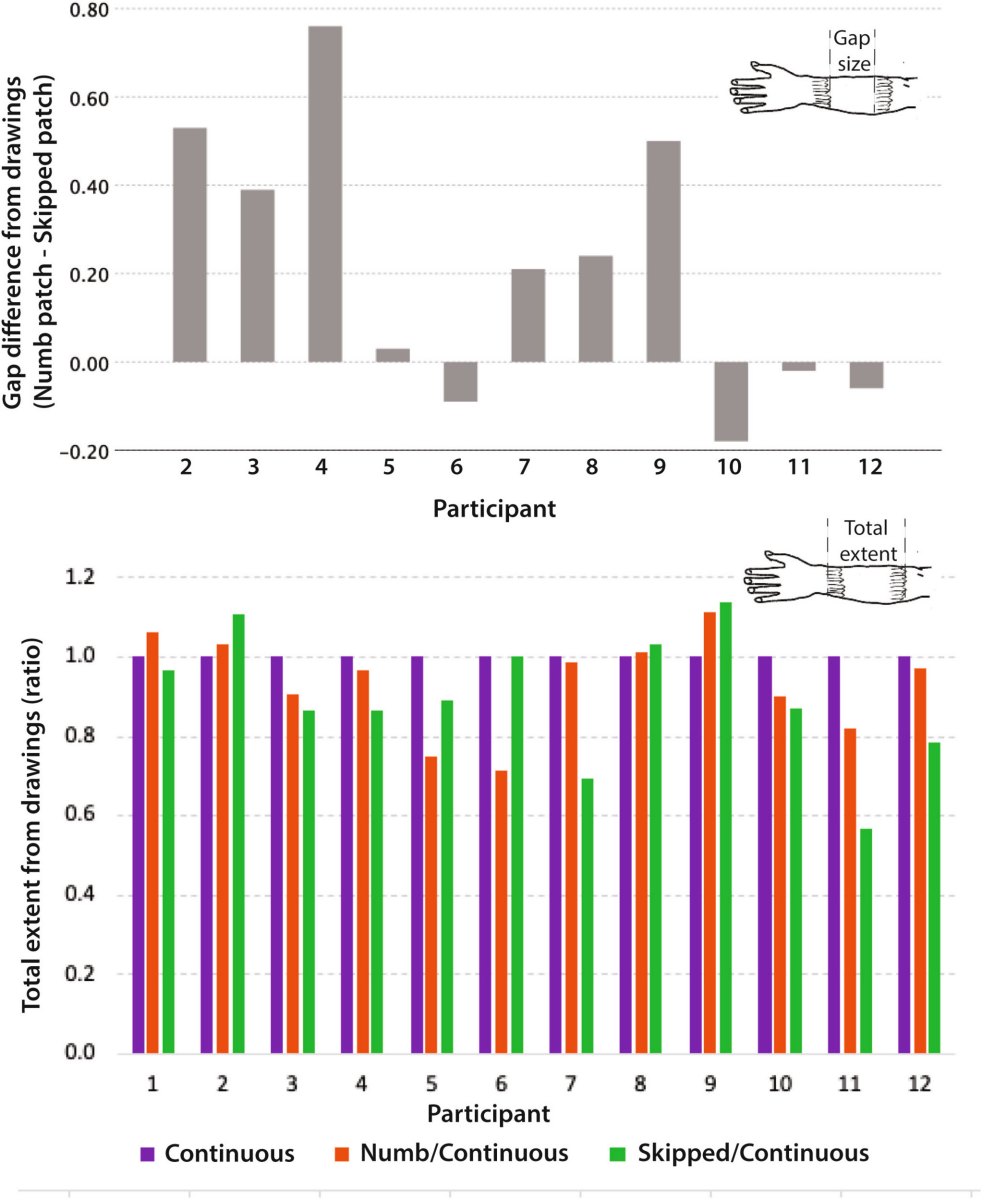
Quantitative results extracted from drawings, individual results. Top: Difference between perceived gap size between Numb patch and Skipped patch (*n* = 11; Participant 1 was excluded from this comparison due to ambiguity in drawings) with gap size expressed relative to the perceived total extent of brushing in each condition (see Figure 8). Bottom: Overall extent of brushed areas as proportions, using Continuous condition as a reference (*n* = 12).

Using bootstrap analysis with 1000 samples (11 participants, excluding P1's ambiguous drawing), we found that the perceived gap was significantly smaller in the Skipped patch compared with the Numb patch condition. The gap size ratio decreased from 0.52 in the Numb patch to 0.31 in the Skipped patch condition (mean difference = 0.21, 95% CI: 0.06–0.40, *p* = 0.049). A paired‐samples t‐test gives a similar result (t(10) = 2.293, *p* = 0.045, mean difference = 0.21, 95% CI: 0.01–0.41). Individual results are shown in the top panel of Figure 9. The smaller gap in the Skipped patch condition suggests perceptual filling‐in occurred when the brush moved quickly over the patch.

The overall extent of the brushed area in participants' drawings for Continuous, Numb Patch and Skipped patch conditions were 33.4 (SD = 4.4), 31.2 (5.0) and 29.8 (5.2) respectively (expressed on an arbitrary scale, in mm as measured on scanned drawings). Individual results are shown in Figure 9, Bottom panel as proportions, using Continuous condition that was typically the longest as a reference.

**FIGURE 10:**
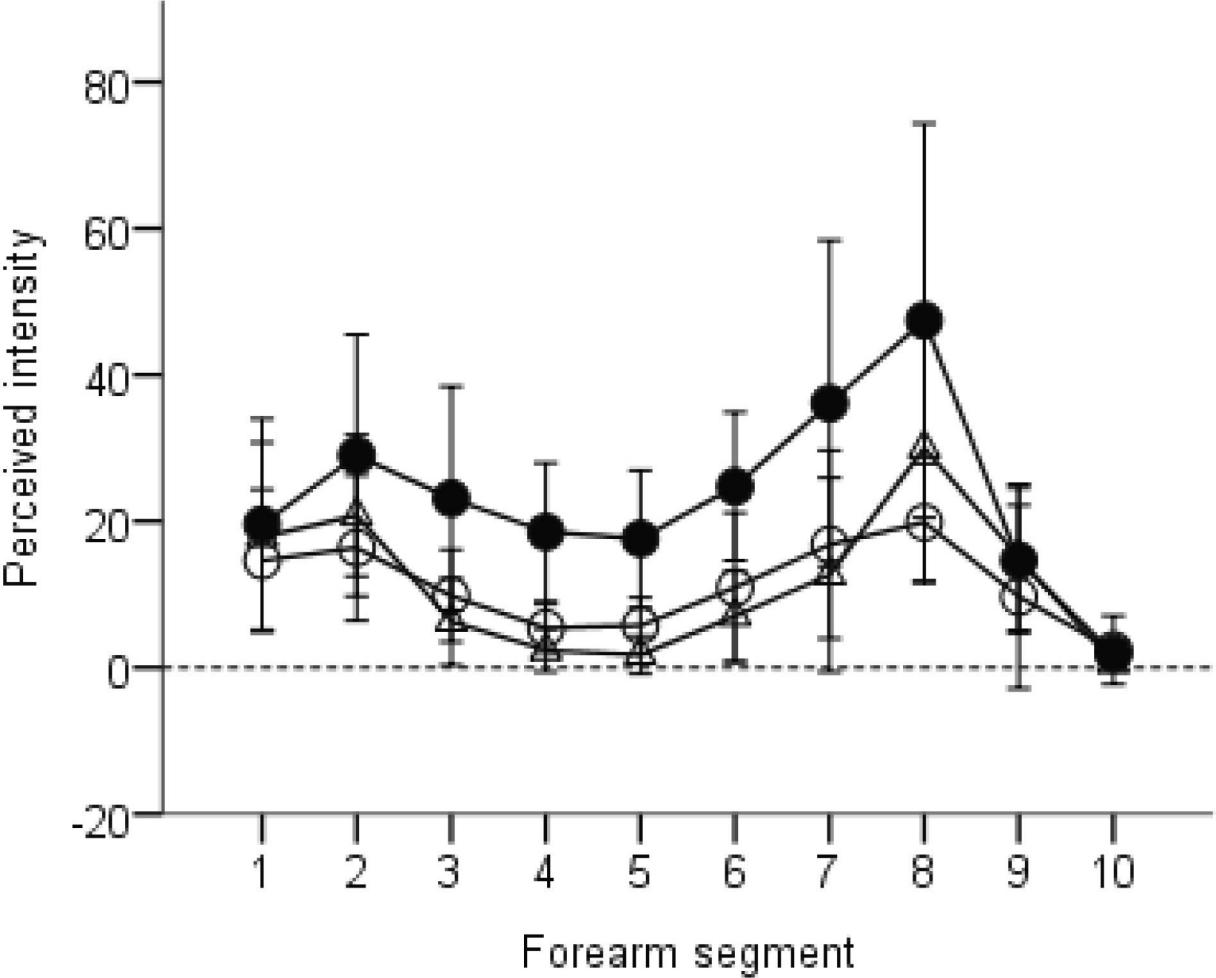
Group means (± 95% CIs, *n* = 12) for perceived intensity of brushing as a function of position along the forearm (1 = near the elbow; 10 = near the wrist) and condition (Continuous, black circles; Numb patch, triangles; Skipped Patch, open circles). Higher values on the y axis represent darker regions in the drawings, that is, more intense perceived brushing.

Intensity levels, averaged across participants for each condition, showed that the Continuous condition had a mean value of 23.3 (± 4.9 standard error), about double that of the Numb patch and Skipped patch conditions, which were 11.4 (± 2.3) and 11.2 (± 1.8), respectively. Perceived intensity peaked near the elbow and wrist, with a decrease in mid‐forearm, across all conditions (see Figure 10).

## 4 Discussion

To explore the role of motion in position coding on the skin, we used a stimulus that moves at a constant speed but skips over a patch of skin without touching it. Acceleration across the gap creates deceptive temporal continuity between skin regions, as if the skipped skin patch does not exist (Seizova‐Cajić and Taylor 2014). Unlike previous studies that examined motion endpoints (e.g., Whitsel et al. 1986; Seizova‐Cajić and Taylor 2014; Nguyen et al. 2016; Macauda et al. 2018; Merz et al. 2019), we tested *post‐motion* localization of *static* stimuli: a von Frey hair applied orthogonally to the skin at 0.5‐cm or 4.5‐cm from the gap edge, 1 s or 10 s after motion offset. This novel approach allowed us to assess whether the influence of motion on spatial perception persists and generalizes to new stimuli, providing stronger evidence for its role in maintaining spatial maps. Localization responses to our von Frey stimuli show an *inward position shift* (Figures 5 and 6). Its magnitude was 4 to 10 mm compared with control conditions that had no gap at all, or no acceleration across the gap. As predicted, accelerated motion across the numb patch led locations on either side to be perceived as closer together. This compression supports our hypothesis that motion continuity helps establish spatial relationships on the skin, with implications for how the nervous system maintains its spatial maps.

Direction of motion prior to localization testing was balanced across trials and delivered in the same manner in the control condition (the numb patch), with the gap approached from both directions; these factors could thus not be responsible for the inward shift observed in the critical condition (skipped patch). Two control experiments (see Supporting Information) ruled out other potential explanations. The first showed that the metal shield used to create the numb patch did not itself cause position bias, addressing concerns about nearby stimuli influencing localization (Day and Singer 1964; Li et al. 2017). The second confirmed that the observed bias was not due to adaptation in tactile sensitivity following brushing.

Phenomenological reports in the form of drawings provided independent confirmation of the compression effect. While participants used different drawing styles to represent their experience (see Results for quantification methods), consistent patterns emerged. The endpoints of brushing were typically drawn near the elbow and wrist, where they were physically located, in all conditions. However, the middle section of the drawings differed with brush speed: the 10‐cm gap in the 19‐cm brush travel was perceived as significantly shorter when the brush accelerated across it (reduced from 52% to 31% of total motion extent). In some cases, the gap was completely absent from perception; it was *completed* or *filled‐in*, and more often so when the brush accelerated than when it did not. Another possibility is that the gap gets ‘stitched‐up’*—*that is, the high‐velocity motion actively brings the skin regions on either side of the gap into perceptual proximity, rather than simply filling in the space between them (for detailed discussion see Seizova‐Cajić et al. 2019).

Our localization and phenomenological results converge to show that acceleration across the numb spot alters spatial perception: both the localization responses and perceived gap size indicate compression towards the middle of the forearm. Since location shifts occurred in the same condition that showed enhanced filling‐in of the gap, common mechanisms may underlie both phenomena. In what follows, we first examine why acceleration leads to position shift of subsequently presented stimuli, and then explore possible links between position shift and filling‐in.

### 4.1 Why Does Acceleration Result in Position Shift of Subsequently Presented Stimuli?

As outlined in Box 1, both continuous and discrete motion can compress perceived spatial relationships in touch, with compression increasing at higher speeds. Most previous evidence comes from judgements of motion endpoints, where participants report locations immediately after motion offset. In contrast, our study shows that motion‐induced compression persists and transfers to new static stimuli presented well after motion ends (up to 10 s post‐motion). The high‐speed motion across the gap appears to create temporary changes in how nearby skin regions are spatially related. Motion‐induced mislocalizations have been attributed to speed priors in Bayesian models (Goldreich and Tong 2013; Merz et al. 2022), while our focus is on how motion continuity creates expectations about spatial relationships. For us, speed changes are a tool used to alter the perceived continuity between skin regions.

Our work is unique in using numb patches to manipulate motion continuity. We proposed (Seizova‐Cajić and Taylor 2014; Seizova‐Cajić et al. 2019, 2020) that when an object moves at constant speed, the nervous system expects this continuity to persist over occluded or numb regions. When this expectation is violated by sudden acceleration, the system responds with a self‐correcting mechanism: it compresses the perceived space between regions to match the temporal proximity created by acceleration. Unlike universal speed priors, this self‐correcting compression reflects a specific response to the immediately preceding motion pattern (for a similar emphasis on immediate context, see discussion of ‘adaptation’ on Merz et al. 2022, 2430).

While the main finding—compressive mislocalization*—*fits our expectation, there were some surprises. When we tested the position of static stimuli post‐motion, we expected this delayed localization task to provide stronger evidence for the role of motion in spatial mapping, but also make the effect harder to obtain. Contrary to these expectations, the position shift was present from the start of each session, remained stable rather than increasing with exposure, and disappeared in the post‐test. This pattern suggests a *fast perceptual mechanism* rather than *slow neuronal accommodation*.

**FIGURE 11:**
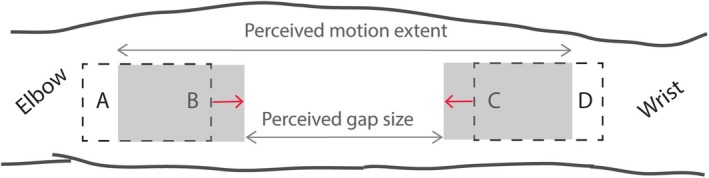
Proposed explanation of position shift. The physical position of brushed areas is indicated by broken outlines, and their perceived position by grey rectangles; the red arrows indicate displacement vectors. High acceleration of the motion stimulus across the gap resulted in gap compression and the two motion fields appeared closer to each other than they were. Subsequently, they were used as reference frames for localization of von Frey stimuli applied at locations A–D, and the displacement vector that applied to the motion fields also applied to those targets. This illustration does not attempt to represent the actual effect size shown in Figure 6. See text for more details.

The velocity‐induced compression described in previous research (e.g., Whitsel et al. 1986) provides a plausible explanation: participants perceived the brushed fields as closer together when the brush accelerated across the gap.^2^ These compressed fields then served as a reference frame for localizing subsequent stimuli, leading to the inward shift we observed (Figure 11).

This compression effect persisted equally at 1 s and 10 s delays, suggesting tactile memory can maintain spatial relationships for at least 10 s in tasks involving a single spatial relationship (compared with typical multi‐item memory tasks; Heled et al. 2021). Recent work shows working memory performance heavily depends on task demands (Cohen‐Dallal et al. 2022). Our participants knew that localization target could be applied 10 s post‐motion, and their sustained attention to the tactile space could have preserved boundaries defined by motion.

The explanation accounts for our key findings: rapid onset, stability across the session, and absence in post‐test. It is also consistent with gap compression from phenomenological reports.

However, participants' drawings present an apparent paradox: while localization showed compression at all test locations including those near elbow and wrist (A, D), the overall motion extent in drawings was not compressed in the skipped patch condition (Figure 8). We propose this reflects the different reference frames used in each task. When drawing at the end of each session, participants likely anchored motion endpoints to anatomical landmarks (elbow, wrist) and no longer relied on motion‐defined boundaries. Supporting this interpretation, experienced observers report that focusing on anatomical landmarks can eliminate perceived compression even during brushing (observation from an unpublished study in which we brushed the index finger).

Another possibility is that participants' entire forearms were perceptually shortened in the skipped patch condition. If so, the compression would not be revealed in drawings since both the forearm and its motion pattern would be proportionally compressed. While we did not test this possibility, it is not an alternative to our main conclusion that acceleration across the gap compresses perceived space but one way the space may get compressed.

### 4.2 Are the Same Neural Mechanisms Responsible for Position Shift and Filling‐In?

Different neural processes may underlie perceptual filling‐in (Pessoa et al. 1998). In our study, gap compression served as an operational measure of filling‐in, which we attribute to motion‐induced shifts of perceived position (Seizova‐Cajić et al. 2019). Supporting this interpretation, accelerating stimuli consistently enhance gap filling‐in across different paradigms (Kaneko et al. 2018; Nguyen et al. 2016; Seizova‐Cajić and Taylor 2014; Seizova‐Cajić et al. 2019). If motion fields indeed shifted, this single mechanism would explain both the filling‐in and subsequent localization bias.

Other filling‐in mechanisms have been identified, primarily in vision but applicable to touch: contextual information bridges gaps through neural gain changes, long‐range connections, and feedback processes (Pettet and Gilbert 1992; DeAngelis et al. 1995; Pessoa and De Weerd 2003; Spillmann et al. 2015; Raman and Sarkar 2016; Carvalho et al. 2021). These mechanisms, which do not require acceleration, could operate through neurons with large receptive fields in areas S1 and S2 (Pei et al. 2011; Costanzo and Gardner 1980) and should have produced filling‐in even in our numb‐patch condition. How acceleration might enhance these processes remains unknown.

**FIGURE 12:**
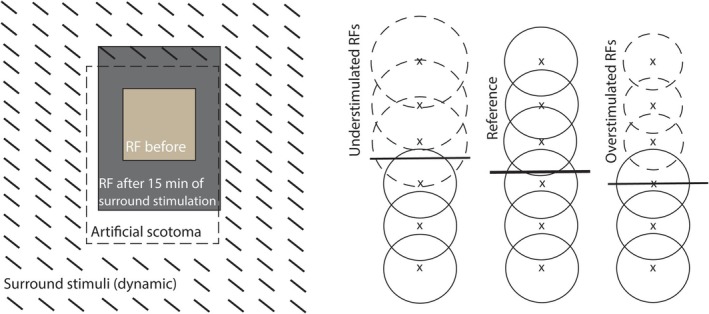
Changes in receptive fields (RFs) of cortical neurons as a possible link between filling‐in and position shift. Left panel: RF of a neuron in cat's V1 (shown in light grey) that initially fell inside the scotoma (indicated by dotted lines) increases after 15 min of surround stimulation to the size indicated by dark grey. Adapted from figure 2 in Pettet and Gilbert (1992). Right panel: Each array depicts a population of neurons that encode position, simplified into a linear array. Perceived position is represented by a horizontal line. The reference state is shown in the middle. Increase in RFs due to lack of stimulation creates attraction bias towards the non‐stimulated area (shown to the left of the reference; adapted from figure 6 in Kapadia et al. 1994), and decrease in RFs due to conditioning, in repulsion bias away from the overstimulated area (shown on the right; see Li et al. 2017). For simplicity, only one boundary between the stimulated and non‐stimulated areas is depicted although long‐range connections would be facilitated by activity on two sides of an artificial scotoma or numb spot; second, gain change is represented spatially in this figure, as expansion or contraction of RFs, although the response increases or decreases throughout the RF.

These same neural mechanisms could also produce position shifts: as illustrated in Figure 12, differential gain changes across neural populations can shift the perceived position (Kapadia et al. 1994; Tailby and Metha 2004, 2006; Li et al. 2017). Since it does not require acceleration, it would also apply to the numb‐patch condition. Two participants showed complete filling‐in even in the numb‐patch condition (P10, P12; see Drawings on OSF), and others may have experienced partial filling‐in (described in vision by Spillmann et al. 2006). However, our design could not fully test for partial filling‐in; future studies could do so by brushing opposite sides of the gap in separate trials (figure 6B in Seizova‐Cajić et al. 2023).

Importantly, while general filling‐in mechanisms would have operated in both the numb‐patch and skipped‐patch conditions, enhanced compression occurred only with fast motion across the gap. This suggests that acceleration‐induced deceptive continuity was the key factor in compressing perceived space between the brushed regions.

### 4.3 Variable Error: Localization Uncertainty

Variable error was consistently smaller near anatomical landmarks (elbow and wrist) than in the middle of the forearm, a pattern widely reported in tactile localization (Boring 1942, describes early work; also see figure 3 in Cholewiak and Collins 2003; figure 5 in Brooks et al. 2019; figure 4 in Miller et al. 2022). This may be due to categorical boundaries between body parts truncating or compressing one side of the error distribution (Huttenlocher et al. 1991). Alternatively, landmarks might serve as reference points for location coding, with noise and thus localization error increasing with distance from the landmark (Miller et al. 2022).

Variable error was also smaller in motion conditions than in post‐tests. This is consistent with our proposal that, in addition to the anatomical boundaries, participants used motion‐defined boundaries to localize subsequent stimuli. It is also possible that the shorter inter‐stimulus interval in the post‐tests led to reduced sensitivity due to the aftereffect from the previous von Frey stimulations.

Importantly, variable error was similar in different motion conditions: it was *not* greater in the critical condition. The similar uncertainty across conditions indicates that Bayesian speed prior accounts (Goldreich and Tong 2013; Merz et al. 2022), which expect greater bias when uncertainty is greater, cannot explain our results through that mechanism. While such accounts could potentially be relevant even without differences in uncertainty, they would need to make specific predictions about how acceleration across a gap leads to systematic compression of perceived space in subsequent localization. Our account proposes that acceleration creates deceptive continuity between regions, compressing the space between them (itself potentially based on prior expectations about what it means to be continuous). This compression then serves as a reference frame for localization*—*a mechanism that could be framed in Bayesian terms but requires more specific explanation than general speed priors.

### 4.4 Future Research

Our proposal that motion fields shifted due to acceleration across the gap can be tested directly by asking participants to continuously track perceived positions of motion fields. Different task instructions would help compare the use of anatomical landmarks versus spatial, object‐centred reference frames.

Our focus has been on acceleration across the numb patch, but in our design this was confounded with average speed of the brush. Continuous motion at a constant speed matching the average speed of our skipped‐patch condition would help determine whether acceleration or average speed is the critical factor for compression.

## 5 Conclusion

Our study extends understanding of how motion influences tactile space perception and potentially somatosensory map plasticity. We demonstrate that motion affects position coding even after it ceases, with effects transferring to new static stimuli applied at locations away from motion endpoints. This paradigm differs from previous studies focused on motion endpoints. Since direction of motion was counterbalanced, it cannot explain the observed position shift*—*only acceleration across the non‐stimulated region can account for this effect. The resulting compression of perceived space remained stable throughout testing, suggesting a fast perceptual mechanism rather than slow map adaptation. Future studies are needed to investigate exposure‐related variations in position coding and link these to specific neural mechanisms.

## Author Contributions

**Tatjana Seizova‐Cajic:** conceptualization, formal analysis, investigation, methodology, supervision, writing – original draft, writing – review and editing. **Jack Brooks:** conceptualization, data curation, formal analysis, investigation, methodology, software, writing – original draft, writing – review and editing. **Janet Taylor:** conceptualization, formal analysis, funding acquisition, investigation, methodology, project administration, resources, supervision, writing – original draft, writing – review and editing.

## Conflicts of Interest

The authors declare no conflicts of interest.

### Peer Review

The peer review history for this article is available at https://www.webofscience.com/api/gateway/wos/peer‐review/10.1111/ejn.70076.

## Supporting information


**Figure S1.** Methods and Results for *Control Experiment 1* (*n* = 8). A. Set up. B. Touch stimuli were delivered at one of the four locations (A, B, C or D) with the sleeve on or with no sleeve. C. Calculated distance between mean responses to outer targets (A, D). D. Calculated distance between mean responses to inner targets (B, C). Dotted horizontal lines indicate the actual distances (18 cm for outer and 11 cm for inner targets). Localization responses were unaffected by the sleeve.
**Figure S2.** Method and individual results for *Control Experiment 2* (*n* = 8). A. Set up:Detection thresholds were tested at one target on the left forearm with one paintbrush moving back‐and‐forth across the target line on the skin of the forearm. B. Touch detection threshold was significantly increased by brushing (*p* < 0.001). C. The weakest intensity at which all touches were felt (100% detection threshold) was also significantly increased by brushing (p < 0.001). Some of the 8 data points overlap because the filament increments are discrete.
**Figure S3.** Variable error across all conditions.
**Figure S4.** Variable error, individual data for motion conditions (top) and post‐tests (bottom). Baseline is shown in both panels for each participant (1 to 12) as blue dotted line. Error bars are 95% CIs.
**Figure. S5.** Variable error as a function of Motion condition, Location and Delay.
**Table S1.** Responses as a function of location and motion condition, estimated marginal means from the Preliminary analysis in which target location was treated as a continuous variable.
**Figure S6.** Responses as a function of target location and order of repeats (there were 8 repeats per condition per participant). Error bars represent 95% CIs. No condition demonstrated a cumulative effect, that is, there was no increased position shift across trials.
**Figure S7.** Localization responses as a function of location along the forearm and motion condition. Each panel shows localization responses from a single participant (P1‐P12). Values represent centered localization responses plotted against centered test locations on the forearm. Same colour code was used as elsewhere: Baseline; Continuous; Numb patch and Skipped patch. Data points represent mean values at each test location based on 32 responses (2 directions of last sweep x 2 delays x 8 repeats), and lines show linear interpolation between adjacent points for each condition.
**Figure S8.** Localization responses as a function of location along the forearm and motion condition. Absolute values of centered localization responses are plotted against target locations on the forearm. Each panel shows localization responses from a single participant.

## Data Availability

The data that support the findings of this study are openly available in Open Science Framework at https://osf.io/4esba/?view_only=e35d88b5e49e4075a20294116459e7ee as per journal policy.

## Endnotes

^1^
Following a reviewer's expression of interest in this, we conducted additional analysis to test the effect of direction of final sweep on the localization response. Three separate LMMs were conducted, one for each motion condition. This revealed a small systematic bias: locations were judged as more distal when the final sweep was proximal compared to distal (mean difference = 0.87 cm [95% CI: 0.64–1.12] in Continuous condition, 0.44 cm [0.22–0.66] in Skipped patch condition, *p* < 0.001 for both). This bias was not significant in the Numb patch condition (0.19 cm [−0.03–0.41], *p* = 0.086). Since the gap was approached from both directions, and direction was counterbalanced, this effect cannot explain our main finding of compression towards the gap.

^2^
Consider Whitsel et al.'s (1986) study, which used brushing like we did. Based on their findings, we might predict halving of the gap when the brush crossed it at the speed of 100 cm^−1^. That would result in approximately 2.5 cm displacement vectors (red arrows in Figure 11), and similar displacements of localization responses, and yet they are much smaller (up to 10 mm, see Figure 6). However, in the Whitsel study, the total length of brushing trajectory was only 4 cm, placed in the middle of the forearm, while our 19‐cm motion trajectory covered most of the forearm. The greater motion extent itself, or the proximity of endpoints to the anatomical landmarks—elbow and wrist—might have resulted in smaller compression in our study.
