# Supplementary material for: Shrinking the Skin: Motion Results in Compressive Mislocalization of Stimuli Applied 10 s Post‐Motion
Source: Eur J Neurosci. 2025 Apr 1;61(7):e70076. doi: 10.1111/ejn.70076 (PMC11962174; doi:10.1111/ejn.70076)
Supplement: Supplementary file 1 — Figure S1. Methods and Results for Control Experiment 1 (n = 8). A. Set up. B. Touch stimuli were delivered at one of the four locations (A, B, C or D) with the sleeve on or with no sleeve. C. Calculated distance between mean responses to outer targets (A, D). D. Calculated distance between mean responses to inner targets (B, C). Dotted horizontal lines indicate the actual distances (18 cm for outer and 11 cm for inner targets). Localization responses were unaffected by the sleeve. Figure S2. Method and individual results for Control Experiment 2 (n = 8). A. Set up:Detection thresholds were tested at one target on the left forearm with one paintbrush moving back‐and‐forth across the target line on the skin of the forearm. B. Touch detection threshold was significantly increased by brushing (p < 0.001). C. The weakest intensity at which all touches were felt (100% detection threshold) was also significantly increased by brushing (p < 0.001). Some of the 8 data points overlap because the filament increments are discrete. Figure S3. Variable error across all conditions. Figure S4. Variable error, individual data for motion conditions (top) and post‐tests (bottom). Baseline is shown in both panels for each participant (1 to 12) as blue dotted line. Error bars are 95% CIs. Figure. S5. Variable error as a function of Motion condition, Location and Delay. Table S1. Responses as a function of location and motion condition, estimated marginal means from the Preliminary analysis in which target location was treated as a continuous variable. Figure S6. Responses as a function of target location and order of repeats (there were 8 repeats per condition per participant). Error bars represent 95% CIs. No condition demonstrated a cumulative effect, that is, there was no increased position shift across trials. Figure S7. Localization responses as a function of location along the forearm and motion condition. Each panel shows localization responses from a single participant (P1‐P12). [file EJN-61-0-s001.docx]

# **Supplementary materials**

[**Supplementary materials** 1](#_Toc188282288)

[Section 1: Control experiments 1](#_Toc188282289)

[Section 2: Variable error in localization responses 5](#_Toc188282290)

[Section 3: Preliminary analysis of position shift 7](#_Toc188282291)

[Section 4: Position shift: individual data 9](#_Toc188282292)

# Section 1: Control experiments

Two preliminary control experiments were conducted to assess whether (1) the constant pressure from a sleeve, as experienced in the main experiment, influenced localization of nearby touch, and (2) the ability to detect touch was influenced by brushing over the skin as applied in the main experiment.

Control Experiment 1 found that localization of touch stimuli was unaffected by the presence of a sleeve. Control Experiment 2 found that both the threshold intensity of touch and the weakest touch that could always be detected were increased after brushing.

Intensity adaptation, leading to a weaker perceived touch post-brushing, could have posed an issue in the main experiment. Weaker stimuli on the forearm tend to be localized more toward the centre of the forearm and with greater variability than stronger stimuli (Brooks et al 2019). However, localization differences are minimal for stimuli well above threshold, across a wide range of intensities. Touches with intensities 5 and 125 times that of the weakest consistently detectable touch were similarly localized (Brooks et al., 2019). Thus, to preclude the effects of intensity adaptation on localization accuracy, the main experiment employed a 60-g von Frey filament. This filament’s intensity is more than six times that of the weakest touch detected post-brushing by any participant from Control Experiment 2.

## Participants

Studies were approved by the University of New South Wales ethics committee and all participants gave written informed consent. All participants were healthy volunteers. Eight people (3F, 5M, aged 22-40), seven right-handed, participated in Control Experiment 1, and eight right-handed people (3F, 6M, aged 21-38) in Control Experiment 2.

## Apparatus and set-up

As in the main experiment, blindfolded participants sat with their left forearm pronated. In addition, graphics tablet parallel to the forearm blocked it from the participant’s view. Touch stimuli were manually applied using von Frey filament. To localise the stimuli, participants pointed to where they felt the touch using a stylus held in the right hand. The position was recorded through the graphics tablet (Fig. S1A).

In Control Experiment 1, the left forearm was fitted with the same leather sleeve that was used in the Numb patch and Skipped patch conditions in the main experiment (shown in Fig. S1A) to the same localization targets (A, B, C and D). However, there was no brushing.

In Control Experiment 2, no sleeve was worn but a paintbrush moved along the arm (Fig. S2). The same carrier moved the brush as in the main experiment, and the same brush was used.

## Control Experiment 1: Pressure from the sleeve and localization

This study investigated whether the skin pressure of the sleeve influenced localization of touch, as previous studies have shown that the simultaneous presence of other touch on the skin influences the spatial perception of a touch ([Braun et al., 2005](#_ENREF_29); Gardner & Spencer, 1972; [Gescheider et al., 1978](#_ENREF_120)).

Touch stimuli were applied by the experimenter using a 60 g von Frey filament at four locations on the forearm, one at a time. These locations were within the windows of the sleeve at ~0.5 cm from the proximal and distal edges of the windows (Fig S1A & B). Participants pointed to the perceived location of touch in two conditions, delivered in two blocks of trials: in one, the sleeve was on the arm (Sleeve), and in the other, it was not used (No Sleeve). In each block, ten stimuli were applied at each of the four locations. They were not preceded by brushing.


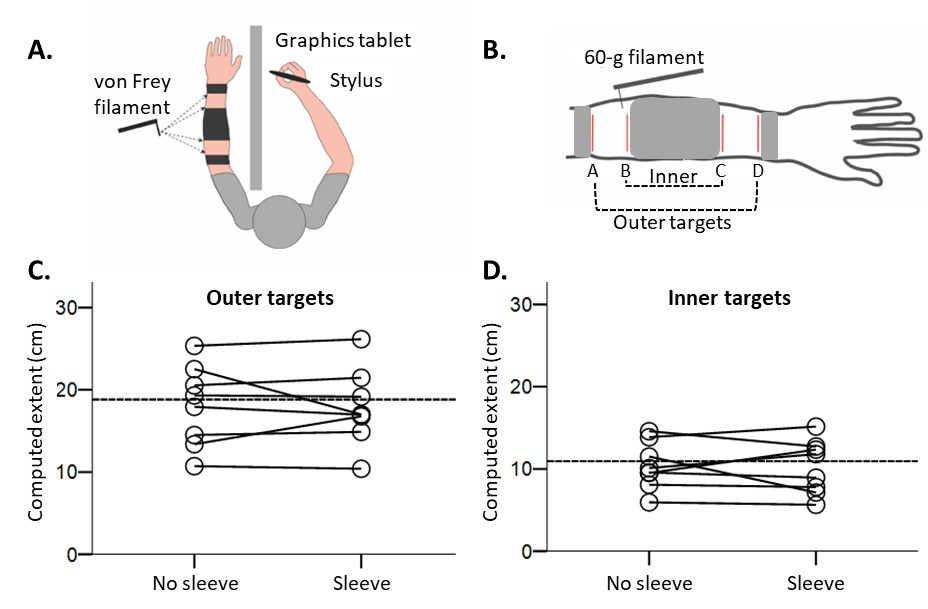


**Figure S1.** Methods and Results for *Control Experiment 1* (n = 8). **A.** Set up. **B.** Touch stimuli were delivered at one of the four locations (A, B, C or D) with the sleeve on or with no sleeve. **C.** Calculated distance between mean responses to outer targets (A, D). **D.** Calculated distance between mean responses to inner targets (B, C). Dotted horizontal lines indicate the actual distances (18 cm for outer and 11 cm for inner targets). Localization responses were unaffected by the sleeve.

For each subject, the mean perceived location of each target location was calculated for each sleeve condition. Then, the distance between the perceived locations of the inner targets was computed, as was the distance between the perceived locations of the outer targets (Fig S1B). Inner targets were 11 cm apart and outer targets were 18 cm apart.

As Fig S1 shows, individual results in the two conditions (Sleeve and No Sleeve) were very similar. Mean computed extents between the outer targets were 17.9 cm (± 4.9) and 17.7 cm (± 4.6), in Sleeve and No Sleeve conditions, respectively; the difference of 0.19 cm tested using paired t-test was not statistically significant (t(7)=0.084; p = .94). For the inner targets, the corresponding values were 10.1 (± 3.3) and 10.3 (± 2.9) cm. The difference of -0.20 cm was also not significant (t(7) = -0.129; p = .90). Variable errors pooled across locations were not significantly different with (12.3 ± 1.0 mm) and without (12.0 ± 0.8 mm) the sleeve (p = .84).

We concluded that the occluding sleeve does not influence the localization of touch stimuli presented adjacent to it.

## Control Experiment 2: Detection of touch before and after brushing

This study measured change in the detectability of touch stimuli due to brushing. If adaptation to brushing reduces the perceived intensity of stimuli, it could influence the results of the main experiment as weak stimuli are localized closer to the centre of the forearm and with more variability than stronger stimuli (Brooks et al 2019).

Only one target location was used in this experiment. Touches were applied at a target line drawn across the forearm, a few centimetres distal to the elbow. During testing of touch, a paintbrush was always in contact with the forearm at a location 7.5 cm distal to the target line (Fig. S2A). Touch detection threshold was measured using a three-alternative forced choice task involving two intervals, announced as ‘first’ and ‘second’. The touch was applied during one of these intervals, and subjects reported whether they felt touch in the first interval, the second interval, or no touch at all.

The forced choice task was delivered in two interleaved staircases. A correct response resulted in use of a weaker von Frey filament for the next stimulus and an incorrect response, a stronger von Frey filament. The termination rule for each staircase was five reversals. Detection threshold was computed as the average of the reversals of the two staircases.

The maximal stimulus intensity for a reversal was then presented ten times to determine whether it was consistently perceived. If subjects made correct detections ten times in ten trials, the intensity was designated suprathreshold. If they made a mistake, the stimulus intensity was increased by use of the next available von Frey filament. This procedure was repeated until 10 correct out of 10 was reached.

Detection thresholds were then remeasured after brushing along the skin of the forearm. First, the brush moved 10 cm up-and-down the arm across the testing line for 4 minutes at a speed of 15 cm/s. After this initial conditioning, six back-and-forth sweeps of the brush were separated by 15-s breaks (no brushing). Threshold and then supra-threshold intensities were determined with touch applied in each 15-s break. The amount of brushing in this control experiment was far greater than in the main experiment, where conditioning consisted of only 1-3 sweeps before each localization task.

Touch detection threshold increased in all participants following the conditioning (see Fig. S2B). The mean detection threshold rose from 0.13 g (SD=± 0.05) to 0.74 g (± 0.10), a change that was statistically significant according to a paired-samples t-test (t(7)=26.98; *p* < .001). The supra-threshold intensity - the weakest intensity that was detected 10 out of 10 times – also showed a significant increase, from 0.50 g (± 0.18) before conditioning to 3.40 (± 0.90) after conditioning (t(7) = 8.94, p < .001; see Fig. S2C).

Conditioning by arm brushing led to significant intensity adaptation. A potential issue is that adaptation might influence localization in the main experiment because touch stimuli would feel weaker after brushing. However, Brooks et al (2019) found that variable and constant errors were comparable across a wide range of strong supra-threshold stimuli. Therefore, employing a stimulus of sufficient strength should mitigate any impact on touch localization resulting from changes in perceived intensity. We used a 60-g von Frey filament in the main experiment. This is more than six times greater intensity than the weakest touch detected 10 out of 10 times after brushing by any participant in this experiment.

**Figure S2.** Method and individual results for *Control Experiment 2* (n=8). **A.** Set up: Detection thresholds were tested at one target on the left forearm with one paintbrush moving back-and-forth across the target line on the skin of the forearm. **B.** Touch detection threshold was significantly increased by brushing (p < .001). **C.** The weakest intensity at which all touches were felt (100% detection threshold) was also significantly increased by brushing (p < .001). Some of the 8 data points overlap because the filament increments are discrete.


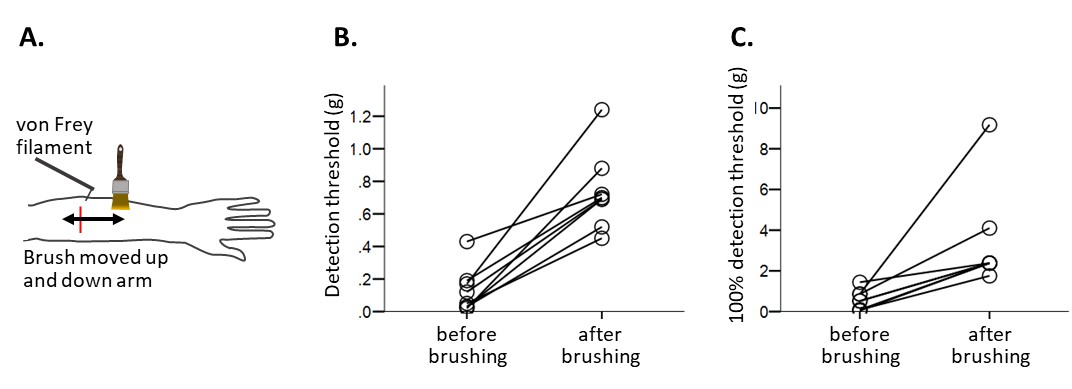


# Section 2: Variable error in localization responses

This section complements related content in the main text with additional graphical data presentations.

Fig. S3 presents data across all conditions, showing that the middle two locations have larger variable error than locations near the elbow (A) or wrist (D). There is a positive skew in the data.

Most individual data, shown in Fig. S4, also follow the quadratic trend. Motion conditions had notably smaller variability than post-tests in all participants. We did not conduct statistical tests to compare the two.

As described in the main text, there was a 3-way interaction between Motion condition, Location and Delay, reflecting the fact that quadratic trends across locations are slightly different at 1 s delay compared to 10 s in different motion conditions. Fig. S5 shows that responses were less variable 10 s post-motion compared to 1 s post motion at locations near the elbow and wrist but not in the middle of the forearm.


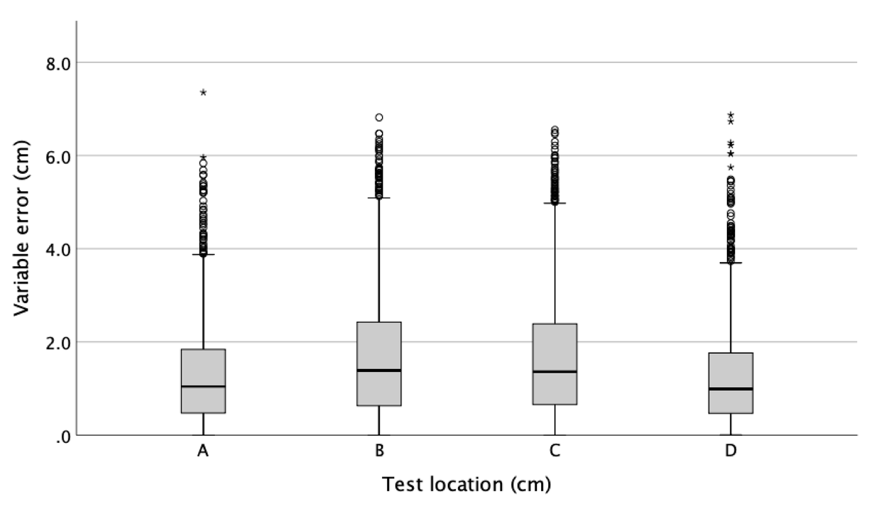


**Figure S3**. Variable error across all conditions.


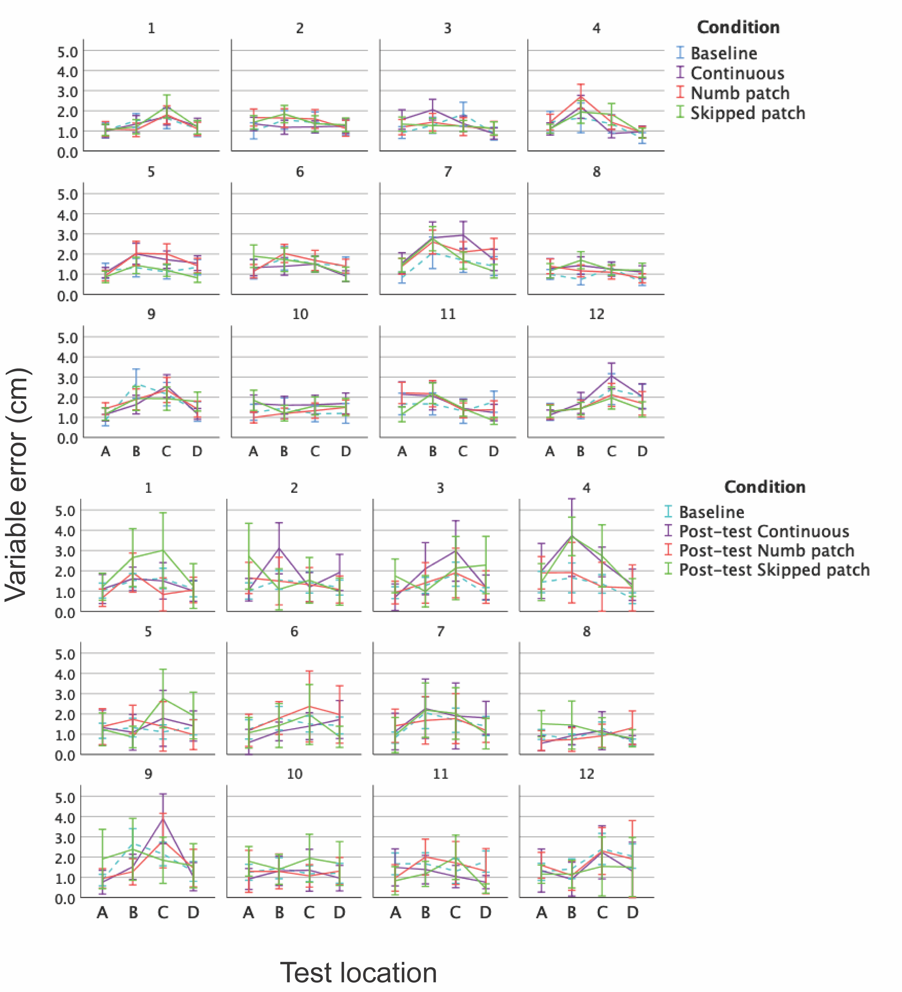


**Figure S4**. Variable error, individual data for motion conditions (top) and post-tests (bottom). Baseline is shown in both panels for each participant (1 to 12) as blue dotted line. Error bars are 95% CIs.

#

# Section 3: Preliminary analysis of position shift


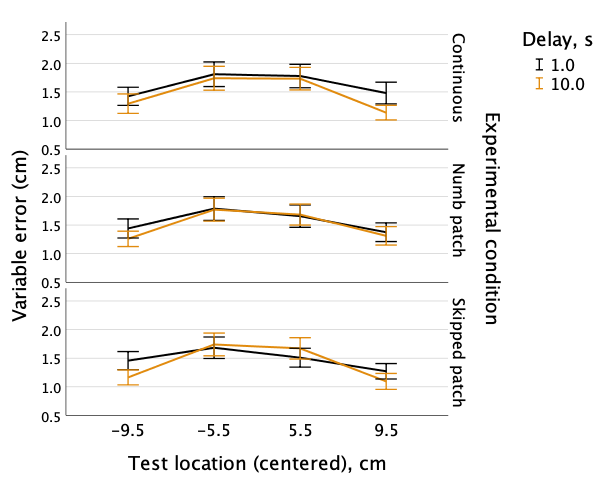


**Figure. S5**. Variable error as a function of Motion condition, Location and Delay

As described in the main text, we performed LMM with Condition (Baseline, Continuous, Numb patch, Skipped patch), Location on the forearm (-9.5, -5.5, 5.5, 9.5) and Order of repeats (1-8) as predictors. Location and Order of repeats were treated as continuous variables. We also tested three 2-way interactions and one 3-way interaction. The full model and detailed SPSS output can be found on [OSF](https://osf.io/4esba/?view_only=e35d88b5e49e4075a20294116459e7ee). Table S1 shows estimated marginal means at each location.

**Table S1**. Responses as a function of location and motion condition, estimated marginal means from the Preliminary analysis in which target location was treated as a continuous variable.

| -9.5 | -5.5 | Target locations | 5.5 | 9.5 |
| --- | --- | --- | --- | --- |
| -8.9 | -5.1 | Baseline | 5.2 | 9.0 |
| -8.6 | -4.9 | Continuous | 5.1 | 8.7 |
| -8.7 | -5.0 | Numb patch | 5.2 | 8.9 |
| -8.1 | -4.6 | Skipped patch | 4.7 | 8.1 |
| -8.6 | -4.9 | Means | 5.1 | 8.7 |

Order of repeats, i.e., the degree of repeated exposure to the motion pattern, was excluded from the model due to a lack of consistent linear trend from the first to the eighth repetition of the stimulus (see Fig. S6). It did not interact with other predictors.

#

**Figure S6**. Responses as a function of target location and order of repeats (there were 8 repeats per condition per participant). Error bars represent 95% CIs. No condition demonstrated a cumulative effect, that is, there was no increased position shift across trials.

#
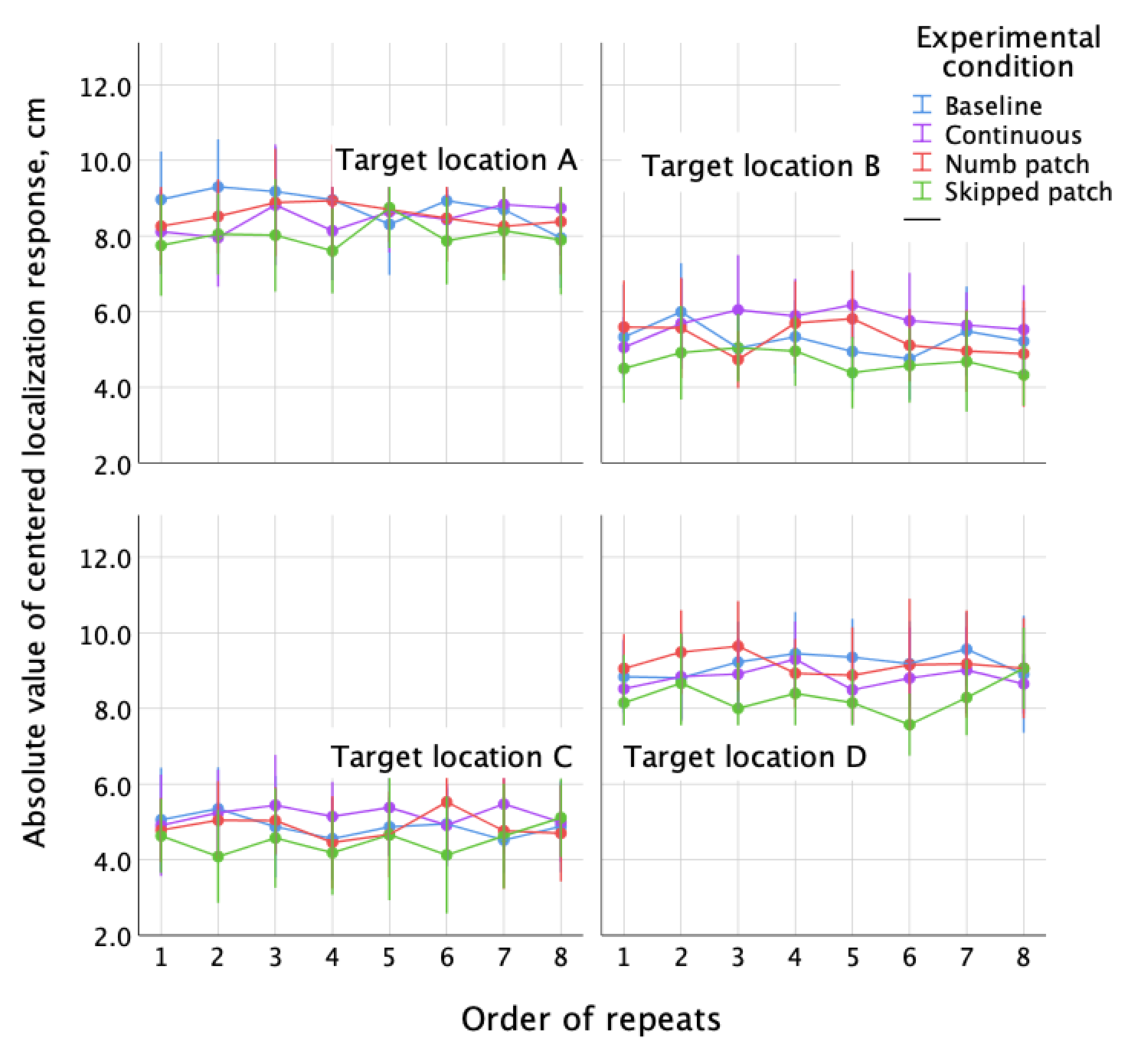
Section 4: Position shift: individual data

Figs. S7 and S8 are a more detailed presentation of the results shown in Fig. 6 in the main text. Individually-centered responses are expressed as absolute distance from the midpoint. The critical condition, Skipped patch, is represented in green. Due to compressive mislocalization, in Fig. S7 it tends to be closer to the midpoint than the other conditions. In Fig. 8, it tends to be the shortest bar.

**Figure S7.** Localization responses as a function of location along the forearm and motion condition Each panel shows localization responses from a single participant (P1-P12). Values represent centered localization responses plotted against centered test locations on the forearm. Same colour code was used as elsewhere: **Baseline**; Continuous; Numb patch and **Skipped patch**. Data points represent mean values at each test location based on 32 responses (2 directions of last sweep x 2 delays x 8 repeats), and lines show linear interpolation between adjacent points for each condition.


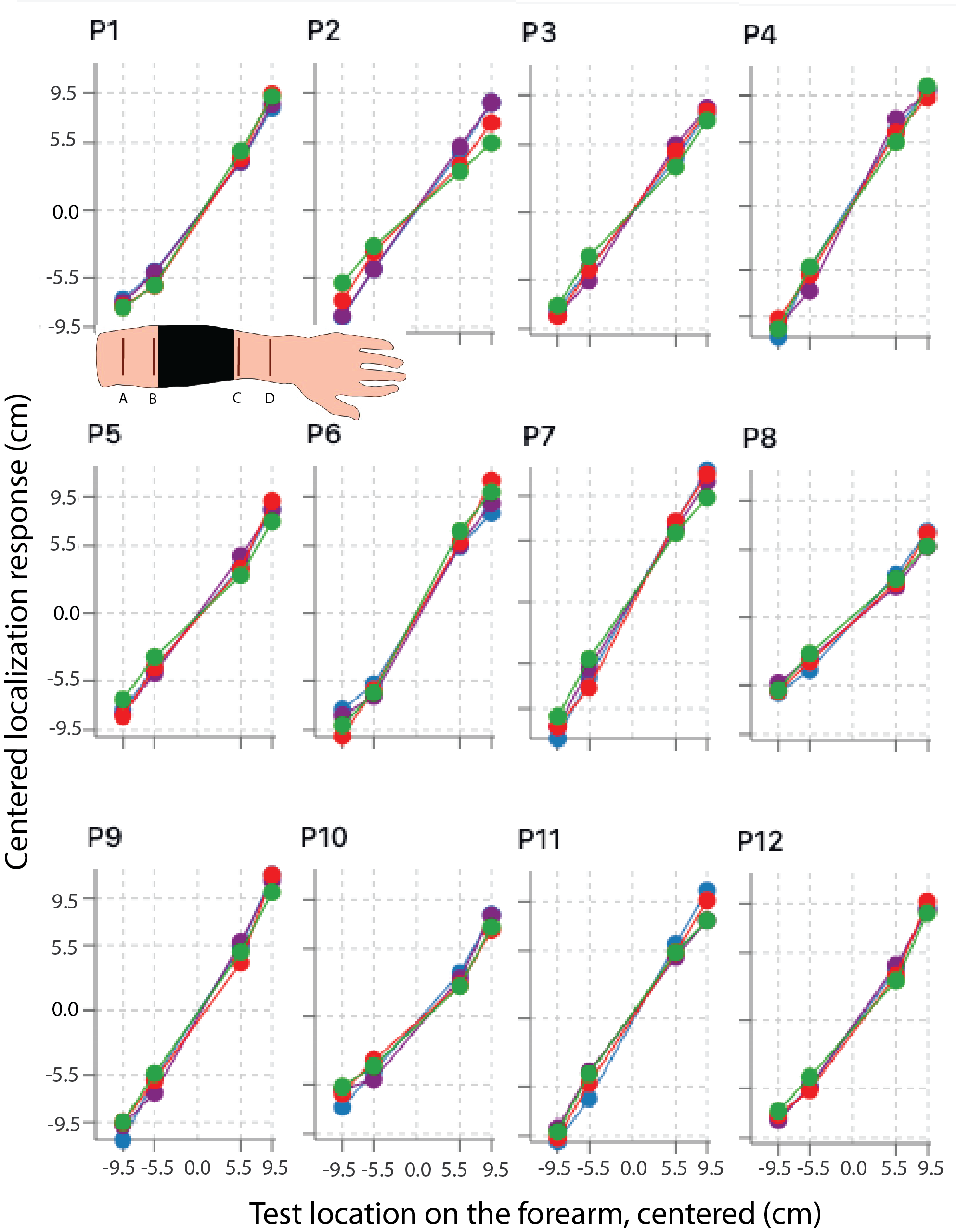


**Figure S8**. Localization responses as a function of location along the forearm and motion condition. Absolute values of centered localization responses are plotted against target locations on the forearm. Each panel shows localization responses from a single participant.


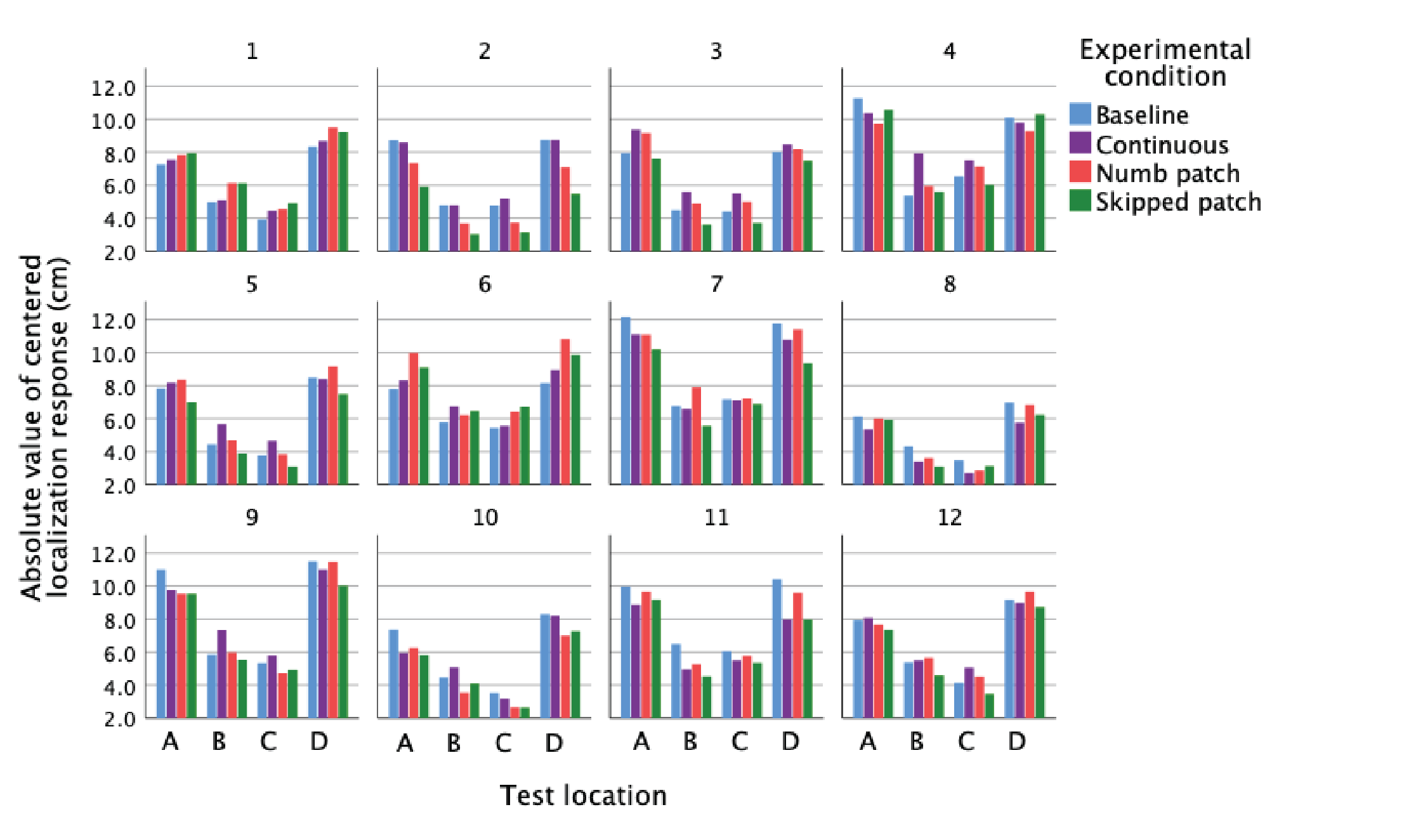


## References

Braun, C., Hess, H., Burkhardt, M., Wuhle, A., & Preissl, H. (2005). The right hand knows what the left hand is feeling. *Experimental Brain Research, 162*(3), 366-373.

Brooks J, Seizova-Cajic T, Taylor JL. (2019) Biases in tactile localization by pointing: compression for weak stimuli and centering for distributions of stimuli. Journal of Neurophysiology. 121(3), 764-772.

Gardner, E. P., & Spencer, W. A. (1972). Sensory funneling: I. Psychophysical observations of human subjects and responses of cutaneous mechanoreceptive afferents in the cat to patterned skin stimuli. *Journal of Neurophysiology, 35*(6), 925-953.

Gescheider, G. A., Capraro, A., Frisina, R. D., Hamer, R. D., & Verrillo, R. T. (1978). The effects of a surround on vibrotactile thresholds. *Sensory Processes, 2*(2), 99-115.
